# Control of intramolecular singlet fission in a pentacene dimer by hydrostatic pressure†

**DOI:** 10.1039/d3sc00312d

**Published:** 2023-02-23

**Authors:** Tomokazu Kinoshita, Shunta Nakamura, Makoto Harada, Taku Hasobe, Gaku Fukuhara

**Affiliations:** a Department of Chemistry, Tokyo Institute of Technology 2-12-1 Ookayama, Meguro-ku Tokyo 152-8551 Japan gaku@chem.titech.ac.jp; b Department of Chemistry, Faculty of Science and Technology, Keio University Yokohama Kanagawa 223-8522 Japan hasobe@chem.keio.ac.jp

## Abstract

Singlet fission (SF), which produces two triplet excitons from a singlet exciton, has been identified as a novel nanointerface for efficient (photo)energy conversion. This study aims to control exciton formation in a pentacene dimer through intramolecular SF using hydrostatic pressure as an external stimulus. We reveal the hydrostatic-pressure-induced formation and dissociation processes of correlated triplet pairs (TT) in SF by means of pressure-dependent UV/vis and fluorescence spectrometry and fluorescence lifetime and nanosecond transient absorption measurements. The photophysical properties obtained under hydrostatic pressure suggested distinct acceleration of the SF dynamics by microenvironmental desolvation, the volumetric compaction of the TT intermediate based on solvent reorientation toward an individual triplet (T_1_), and pressure-induced shortening of T_1_ lifetimes. This study provides a new perspective on the control of SF by hydrostatic pressure as an attractive alternative to the conventional control strategy for SF-based materials.

## Abstract 2

We investigated singlet fission (SF) effects of a biphenyl-bridged pentacene dimer upon hydrostatic pressurization, revealing that the desolvation process in polar solvents plays an important role in the SF dynamics under high pressure.

## Graphical abstract

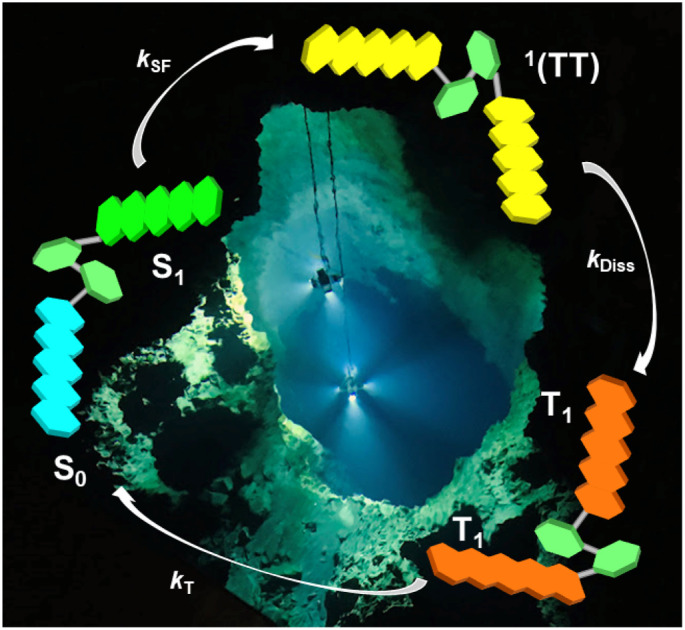

## Introduction

**Fig. 1:**
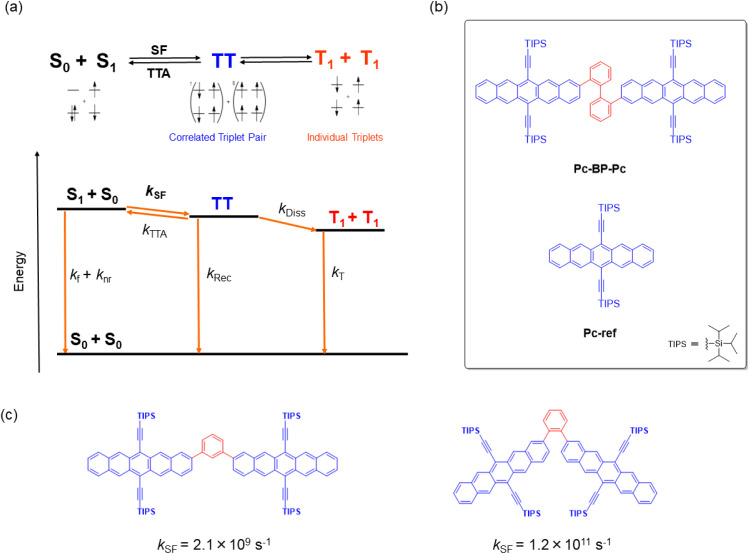
(a) Schematic of the mechanism of SF in a TIPS-pentacene dimer. *k*_f_ and *k*_nr_: deactivation processes from S_1_; *k*_SF_: SF from S_1_ to TT; *k*_Rec_: recombination process from TT to S_0_; *k*_Diss_: dissociation process from TT to T_1_ + T_1_ (2T_1_); *k*_T_: deactivation process from 2T_1_ to S_0_. Considering the reported energies of S_1_ (1.9 eV) and TT (1.6 eV), the reverse triplet–triplet annihilation process (TT → S_0_ + S_1_) can be omitted because of the large exergonic trend (∼0.3 eV).^42^ (b) Target chemical structures of Pc-BP-Pc and Pc-ref. (c) Structures and their SF rates of TIPS-pentacene dimers that are linked by *m*- and *o*-phenylenes.^42^

Singlet fission (SF) is a reversible photophysical process in which two chromophores in the ground state (S_0_) and an excited singlet state (S_1_) interact to form correlated triplet pairs (^1^(TT) and ^5^(TT)). These pairs then relax, forming two individual excitons (T_1_) with an extremely high T_1_ quantum yield (*Φ*_T_) of up to 2 (Fig. 1a).^1–3^ This process is very promising and even more successful than other recently developed photophysical processes, including thermally activated delayed fluorescence (TADF),^4^ aggregation-induced emission (AIE),^5^ and upconversion,^6^ toward the construction of photo-relevant materials and optical chemosensors.^7–9^ For example, the highly efficient generation of multiple excitons allows for a wide array of applications for SF-based materials, including the construction of solar cells,^10^ photosensitizers,^11^ singlet oxygen generators for photodynamic therapy, and relevant biological systems.^12^ Nevertheless, the fabrication of SF-based materials requires that the important energy balance at the excited singlet and triplet levels be met (*E* (S_1_) ≥ 2*E* (T_1_)),^1–3^ which in turn can hamper their wide range of molecular design. One appropriate solution for adjusting such rigidity or non-tunability of SF properties is the flexible/dynamic control achieved by external stimuli such as temperature,^13–16^ solvent,^17–19^ or reaction (aggregation^20–23^ and supramolecular complexation^19,24,25^). This control strategy has recently attracted significant attention although it is yet to be demonstrated on a trial-and-error basis.

Hydrostatic pressure is a mechanical isotropic stimulus and one of the most significant state quantities, enabling scientists to precisely control thermodynamic equilibria and kinetic rates, particularly in solutions in both ground and excited states.^26–28^ Thus, studies on hydrostatic pressure have been widely conducted since the early 1960s.^29^ In the 1980s, advances were made in the understanding of photophysical and photochemical characteristic processes, such as excimer/exciplex formation,^30,31^ photoinduced electron transfer,^32^ and twisted intramolecular charge transfer^33^ upon hydrostatic pressurization. In this way, the effects of hydrostatic pressure on the properties of solutions have been investigated on a long-term basis, in addition to solid-state high-pressure chemistry using a diamond anvil cell (DAC).^34,35^ Although such high-pressure chemistry has been extensively researched, recently, the emergence of mechanochemistry^36^ and mechanobiology,^37^ in which “pressure” as a mechanical force plays a key role, has brought solution-state hydrostatic pressure chemistry under the spotlight once again. We recently revealed that the TADF properties in a mechanochromic material^38^ and the on–off AIE behavior of dynamic AIE-active polymers^39^ can be controlled by the application of hydrostatic pressure. These results prompted us to examine how hydrostatic pressure affects or “controls” the SF processes in functional molecules.

In this study, to implement the hydrostatic pressure control concept in SF chemistry, we focused on a biphenyl-bridged pentacene dimer (Pc-BP-Pc, Fig. 1b), which possesses a more flexible biphenyl linker than its corresponding reference monomer (Pc-ref, Fig. 1b). Recently, Asbury *et al.* investigated the solid-state pressure effects of Pc-ref using the DAC technique, revealing efficient triplet-pair separation.^40^ This sophisticated case provided us with a breakthrough in the design of smart solar cells and relevant materials in the thin film state. However, control of solution-state SF processes upon the application of hydrostatic pressure, for example, photodynamic therapy in physiological (buffer) solutions, remains a major challenge in current multidisciplinary chemistry.

As a guideline for molecular design, the choice of linkers between bichromophores^41^ is significant for achieving the stated purpose. This significance is highlighted by the results shown in Fig. 1c: the SF kinetics (*k*_SF_) in the *m*- and *o*-phenylene bridges in toluene were 2.1 × 10^9^ s^−1^ and 1.2 × 10^11^ s^−1^,^42^ respectively, indicating that adjusting the distance and angle of each chromophore is critical. These results allowed us to choose a relatively flexible biphenyl linker with a *k*_SF_ of 1.8 × 10^9^ s^−1^ in tetrahydrofuran (THF) under atmospheric conditions (0.1 MPa).^17^

We now report the unprecedented excited-state dynamics of Pc-BP-Pc, in which the SF kinetics are drastically facilitated by hydrostatic pressurization. This study enabled us to examine the extent to which hydrostatic pressure affects the SF dynamics. The present demonstration of hydrostatic pressure control highlights the potential for a wide variety of attractive applications using SF processes in solution systems.

## Results and discussion

### Investigation of pressure-induced structural relaxation

First, to investigate the degree of aggregation of the pentacene dimer under hydrostatic pressure (∼320 MPa), concentration-dependent (14–228 μM) UV/vis spectra were measured at 0.1 (atmospheric pressure), 160, and 320 MPa in toluene. As shown in Fig. S2 in ESI,† the absorbance increased at a constant rate with increasing concentration without any spectral changes in each hydrostatic pressure range, with the standard absorbance curves exhibiting strong linear relationships. Therefore, we confirmed that pressure-induced aggregation and crystallization based on intermolecular interactions of Pc-BP-Pc do not occur in these concentration ranges, which enables us to treat the pentacene analogs as well-dispersed, “monomeric” states in the following experiments.

**Fig. 2:**
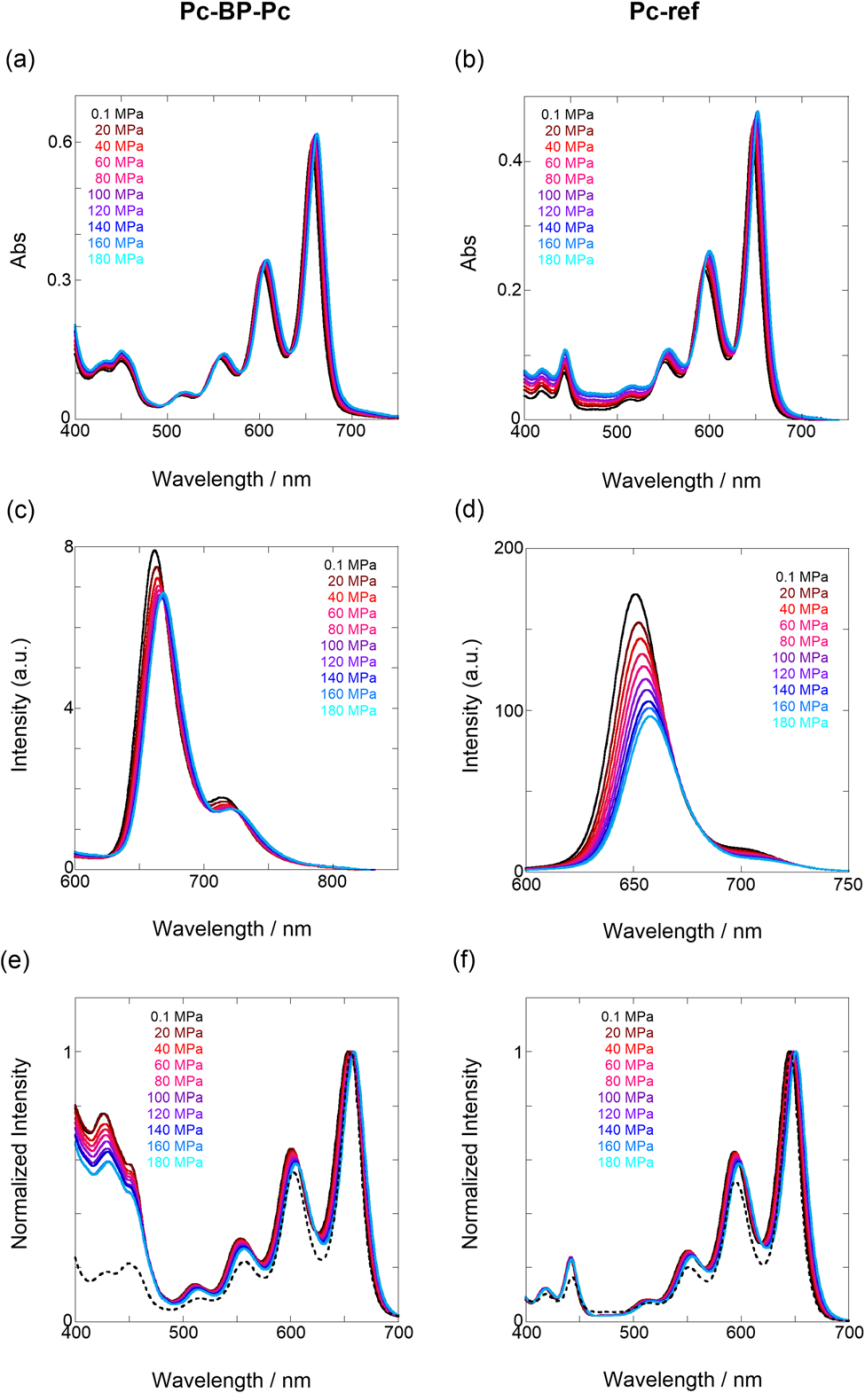
(a–f) Pressure-dependent UV/vis spectra of (a) Pc-BP-Pc (82 μM) and (b) Pc-ref (91 μM); fluorescence spectra of (c) Pc-BP-Pc (67 μM) (*λ*_ex_ = 580 nm) and (d) Pc-ref (91 μM) (*λ*_ex_ = 543 nm); and excitation spectra of (e) Pc-BP-Pc (82 μM) (*λ*_em_ = 660 nm) and (f) Pc-ref (91 μM) (*λ*_em_ = 650 nm) in toluene at room temperature at 0.1, 20, 40, 60, 80, 100, 120, 140, 160, and 180 MPa (from black to sky blue), measured in a high-pressure cell. The black dotted lines represent the normalized UV/vis spectra at 0.1 MPa.

Next, through steady-state UV/vis and fluorescence spectrometry under hydrostatic pressure, we investigated the effect of hydrostatic pressure on both ground-state absorption and excited-state fluorescence properties. As a control experiment, we performed similar tests using Pc-ref, a monomeric portion of the target pentacene dimer, and compared the pressure-induced spectral changes. As shown in Fig. 2a–f, under hydrostatic pressure, the spectra of both Pc-BP-Pc and Pc-ref showed gradual increases in absorbance and stepwise bathochromic shifts without significant changes in the spectral shape. This result suggests that intramolecular π-stacking does not occur in the ground state and in the excimer species in the excited state even under high pressure. It is well known that an increase in pressure induces a considerable change in solvent polarizability, causing the absorption and fluorescence peaks to decrease in energy.^29^ In addition, the monotonic hyperchromic effect on absorbance is simply due to the increase in the effective concentration upon pressurization. Therefore, such pressure-induced wavelength shifts can provide us with significant information about the pentacene analogs in hydrostatic-pressurized solutions; the absorption and fluorescence slopes in toluene were calculated to be −0.750 and −0.856 cm^−1^ MPa^−1^ for Pc-BP-Pc, and −0.848 and −0.891 cm^−1^ MPa^−1^ for Pc-ref, respectively, as listed in Table S1.† These values appear to be very similar to those observed in other π systems such as anthracene, pyrene, and perylene.^28^ Hence, these results confirm that the intramolecular stacking behavior (*vide supra*) and substantial conformational relaxation around the biphenyl linker (particularly in the S_1_ transition) may not be affected by hydrostatic pressure. Parallel experiments in methylcyclohexane (MCH) and THF showed similar slopes from −0.588 to −0.755 cm^−1^ MPa^−1^, verifying the occurrence of no significant conformational changes even in a wide variety of dipole moments (0.00–1.75 (Table S1 and Fig. S3–S10 in ESI†)).

### Intramolecular SF dynamics

**Fig. 3:**
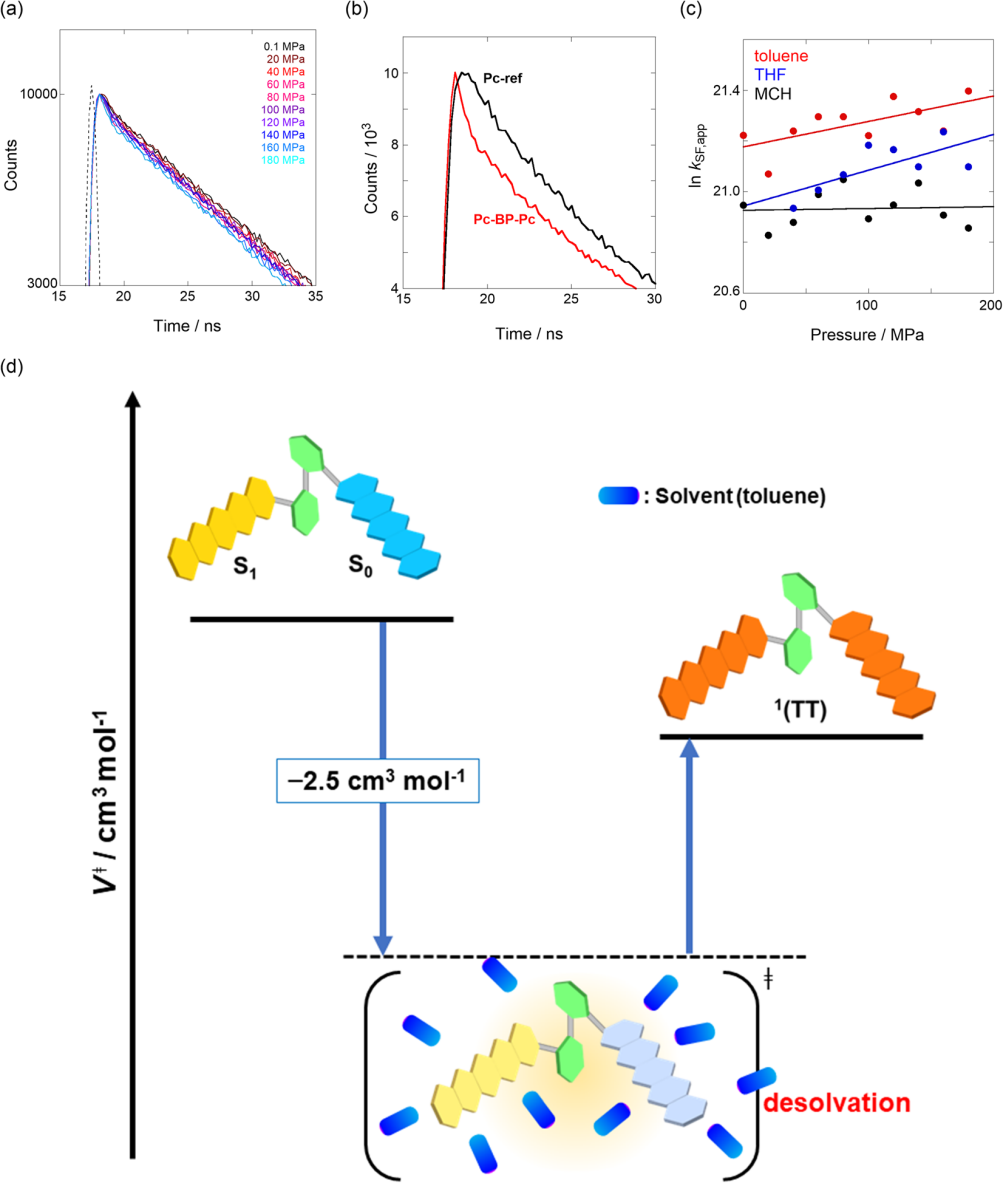
(a and b) Enlarged time-correlated fluorescence decays (*λ*_ex_ = 405 nm) of (a) Pc-BP-Pc (74 μM) (*λ*_em_ = 660 nm) in toluene at room temperature at 0.1, 20, 40, 60, 80, 100, 120, 140, 160, and 180 MPa (from black to sky blue) and (b) Pc-BP-Pc (74 μM) (*λ*_em_ = 660 nm, red) and Pc-ref. 89 μM (*λ*_em_ = 650 nm, black) in toluene at room temperature at 180 MPa, measured in a high-pressure cell. The black dotted line represents the instrument response function. (c) Pressure dependence of SF rate constants (*k*_SF,app_) of Pc-BP-Pc in toluene (red, correlation coefficient *r* = 0.655), THF (blue, *r* = 0.706), and MCH (black, *r* = 0.059) at room temperature; the separate plots with error bars are given in Fig. S26.† The large deviations for *r* are simply based on the roughness of the number of the digits in the lifetime apparatus. (d) Schematic diagram of Pc-BP-Pc for volumetric changes in the transition state *via* the intramolecular SF process.

We estimated the effects of hydrostatic pressure on the intramolecular SF kinetics of the pentacene analogs. The fluorescence lifetimes of Pc-BP-Pc and Pc-ref under hydrostatic pressure were measured in toluene (Fig. 3a and b), with the resulting decay profile obtained for Pc-BP-Pc containing multiple components. The profile reasonably fitted to the sum of two exponential functions, in contrast to that of the monoexponential function observed for Pc-ref (see Fig. S11–S16 and Tables S2–S4 in ESI†). Very short-lived decays (*τ*_2_) in Pc-BP-Pc are observed in the enlarged figure. The flexible Pc-BP-Pc dimer adopts some conformers,^17,43^ in which the *τ*_1_ species emits the fluorescence as a monomer (without the SF process) and the *τ*_2_ species mainly decays as a deactivation path (involved the SF process). Indeed, the strong fluorescence quenching of Pc-BP-Pc at 0.1 MPa, rather than Pc-ref, was clearly observed.^17^ Certainly, other Pc dimers also showed the same decay behavior; the long-lived species (monomer conformer) and the short-lived species (TT process).^44^ Thus, the rate constant of SF, *k*_SF,app_, for the generation of correlated TTs can be written as eqn (1):1
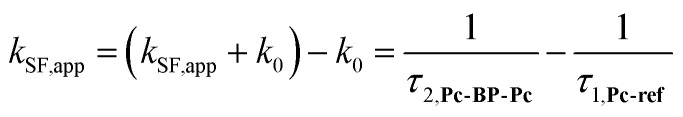
where *k*_0_ represents the decay rate constant from the excited singlet state Pc-ref, which can further be divided into the sum of *k*_f_ (fluorescence rate constant) and *k*_nr_ (radiationless deactivation rate constant). According to our previous study,^17^ the rate constant in the SF process should be expressed *k*_SF,app_ as an apparent rate because the reverse TTA process has not been proved in this dimer. As listed in Tables 1 and S5,† the *k*_SF,app_ values increased from 1.65 × 10^9^ s^−1^ to 1.96 × 10^9^ s^−1^, consistent with the elevated pressures (0.1–180 MPa) in toluene. Interestingly, a clear increasing trend of *k*_SF,app_ from 1.23 × 10^9^ s^−1^ to 1.67 × 10^9^ s^−1^ (40–160 MPa) in the more polar THF upon hydrostatic pressurization was also observed, whereas the *k*_SF,app_ values in nonpolar MCH were almost constant in the range of pressures examined. These findings indicate that hydrostatic pressure critically affects the SF process with solvent polarity.

**Table 1:** Apparent rate constants of singlet fission (*k*_SF,app_) and activation volume changes (Δ*V*^‡^) of Pc-BP-Pc under hydrostatic pressure^a^

Solvent	Dipole moment/*D*	*k*_SF,app_/10^9^ s^−1^				Δ*V*^‡^/cm^3^ mol^−1^
		0.1 MPa	60 MPa	120 MPa	180 MPa	
MCH	0.00	1.25	1.30	1.25	1.14	−0.2 ± 1.1
Toluene	0.38	1.65	1.77	1.92	1.96	−2.5 ± 1.0
THF	1.75	b	1.33 (1.23)^c^	1.56	1.45 (1.67)^d^	−3.5 ± 1.4

a Measured at 298 K.

b Not determined.

c At 40 MPa.

d At 160 MPa.

To further elucidate the hydrostatic-pressure-induced SF dynamics more quantitatively, we calculated the activation volume change (Δ*V*^‡^) in the transition state according to eqn (2):2
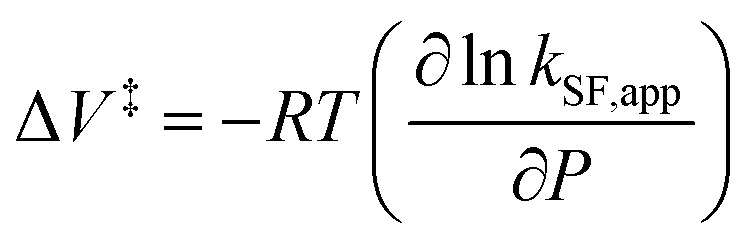


The natural logarithm of *k*_SF,app_ was plotted against pressure, with a linear relationship obtained in each solvent (Fig. 3c). This indicates that the excited-state reaction toward the transition state proceeds by only a single mechanism, that is, the SF process, in the range of the hydrostatic pressures examined. A distinct difference in the negative Δ*V*^‡^ was observed for the different solvents: −2.5 cm^3^ mol^−1^ in toluene and −3.5 cm^3^ mol^−1^ in THF, compared to the almost zero Δ*V*^‡^ (−0.2 cm^3^ mol^−1^) in MCH (see Table 1). Considering that Δ*V*^‡^ is the degree of compactness in a transition state depending on not only structural changes, such as differences in bond lengths and angles,^26^ but also solvation,^28,45^ in this case relating to the intramolecular SF system, the latter solvation contribution seems to be preferable. Hence, these results can be explained in terms of the intramolecular charge density change in the correlated TT. As the desolvation process proceeds, the transition-state structure becomes more compact in polarized toluene (0.38 D) or THF (1.75 D), as shown in Fig. 3d. This scenario may be supported by a very small contribution of Δ*V*^‡^ in nonpolarized MCH (0.00 D). Here, as the volume of one toluene or THF molecule is 106 cm^3^ mol^−1^ or 81 cm^3^ mol^−1^, the desolvation contribution to each Δ*V*^‡^ is approximately 2% in one toluene molecule or 4% in one THF molecule. This indicates that intramolecular SF processes can be accelerated by critical desolvation, which allows the transition-state assembly involving the solvent cluster to be much more compact. Therefore, the solvation/desolvation contribution plays a decisive role in the precise control of intramolecular SF dynamics.

### Hydrostatic pressure effects of triplets

**Fig. 4:**
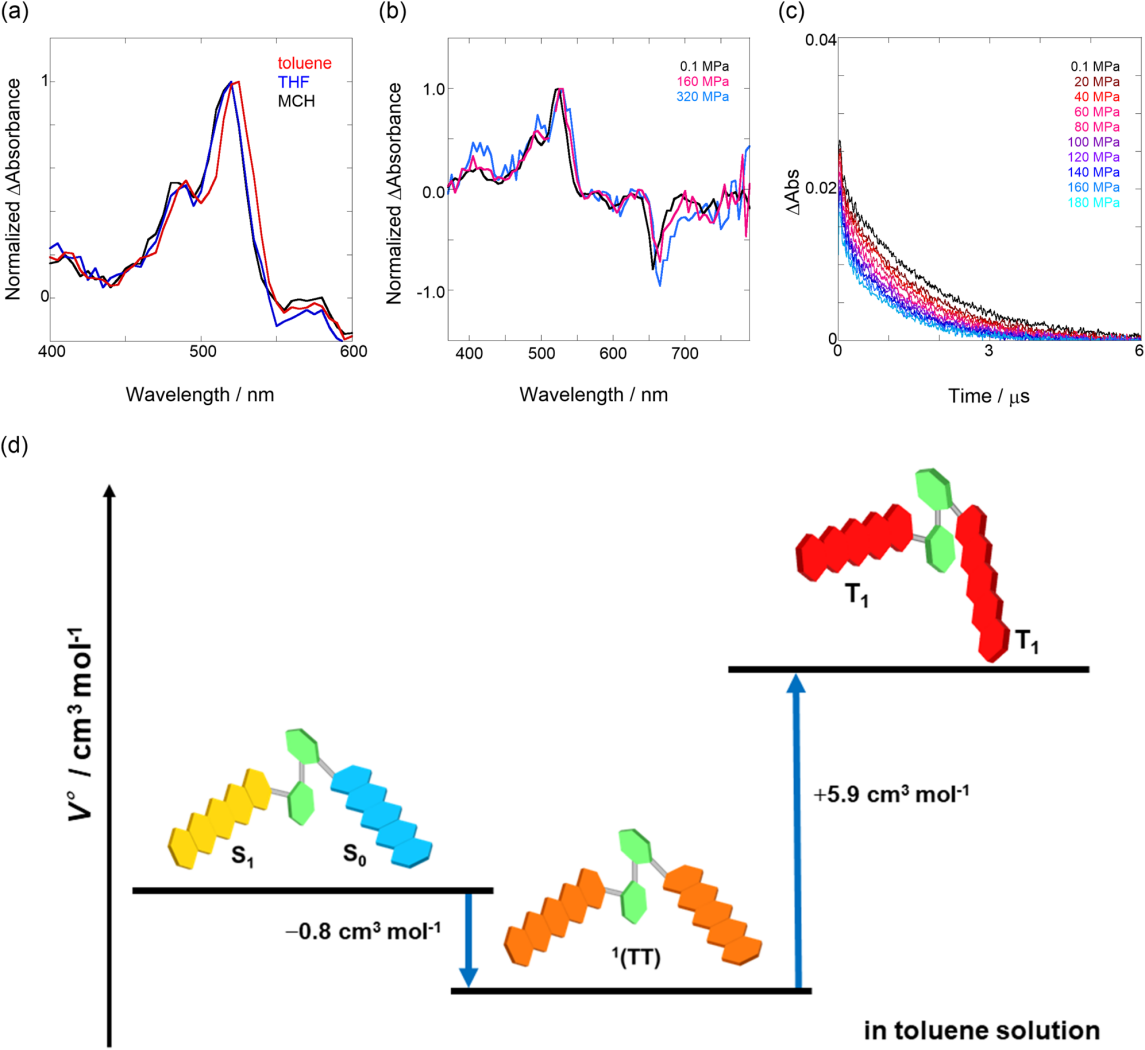
(a and b) Normalized nsTA spectra of Pc-BP-Pc (81 μM) in (a) toluene (red), THF (blue), and MCH (black) at 0.1 MPa and (b) toluene at 0.1 (black), 160 (pink, the line at 510–520 nm was omitted due to an artifact), and 320 MPa (blue) at room temperature. (c) nsTA decay profiles of Pc-BP-Pc in toluene (81 μM, *λ*_ex_ = 532 nm, *λ*_obs_ = 521 nm) at 0.1, 20, 40, 60, 80, 100, 120, 140, 160, and 180 MPa (from black to sky blue). (d) Schematic of the volumetric changes in the equilibria *via* the intramolecular SF and dissociation processes for Pc-BP-Pc.

We elucidated the hydrostatic-pressure-induced intramolecular SF processes, at which the active exciton, TT, eventually becomes T_1_. Nanosecond transient absorption (nsTA) spectrometry is an effective analytical tool for elucidating the photophysical properties of T_1_ as a final step of SF.^17^ The construction of a newly designed optical system for hydrostatic pressure nsTA spectrometry is detailed in the Materials and methods section. This system was used to measure the solvent-dependent spectra of toluene, MCH, and THF solutions in the triplet absorption band (Fig. 4a). As shown in Fig. 4b and S17a–c,† spectral measurements at 0.1 (atmospheric pressure), 160, and 320 MPa also showed pressure-dependent decays and slight pressure-induced bathochromic shifts, similar to those observed in the steady-state UV/vis absorption spectra upon hydrostatic pressurization. In addition, the nsTA decay profiles of the generated individual T_1_ at each hydrostatic pressure in Fig. 4c and S17d† can be reasonably fitted to a monoexponential function with triplet lifetimes (*τ*_T_) of 0.7–1.6 μs (Fig. S18–S19 and Table S6†). So far, we have revealed that *τ*_T_ of Pc-BP-Pc can be prolonged from 0.36 μs in THF to 1.0 μs by mixing paraffin into THF, based on the suppression of collisional deactivation by the polar solvent.^17^ We achieved precise hydrostatic pressure control of *τ*_T_ without changing the solvent, in which the shortened lifetimes in response to hydrostatic pressurization are responsible for T_1_ deactivation under higher viscosity solvent conditions, that is, acceleration of solvent interactions with T_1_ excitons (*vide infra*: result of the bulkier T_1_ structure). As shown in Fig. S27,† the pressure-induced viscosity changes play significant roles in the excited-state processes rather than the polarizability, density, and polarity; the latter ones almost affect to the absorption behavior.^29^ Indeed, Lacour and Vauthey *et al.* demonstrated the SF control by solvent viscosity.^19^

Finally, the S_1_, TT, and T_1_ quantum yields (*Φ*_S_, *Φ*_TT_, and *Φ*_T_) were calculated (see the Materials and methods section), enabling us to estimate the SF thermodynamics, that is, the reaction volume changes as 
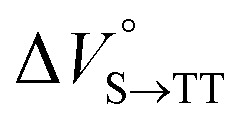
 in the equilibrium between S_1_ and TT (eqn (3)) and 
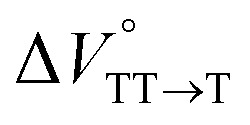
 in the equilibrium between TT and T_1_ (eqn (4)).3
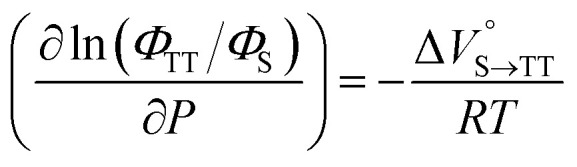
4
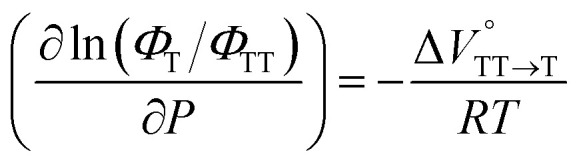


As shown in Table 2 and Fig. S22,† the values of 
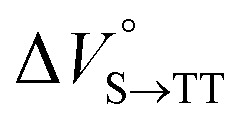
 in toluene and MCH were negative and almost zero, respectively, indicating that the TT structure comprising the solvent core was thermodynamically more compact than the S_1_ structure upon desolvation. This is consistent with the fact that the kinetically formed TT transition-state complex is also more compact owing to the desolvation-driven behavior (*vide supra*). In contrast, the values in Table 2 and Fig. S24† for 
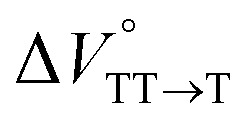
 were positive. Strangely, these values in both toluene and MCH are approximately +5.8 cm^3^ mol^−1^; such a solvent-independent trend suggests that a different mechanism is at play here, rather than the aforementioned solvation/desolvation. These results are likely attributable to the change in conformers in Pc-BP-Pc during the TT dissociation process (TT → 2T_1_), as shown in Fig. 4d. In a previous study on the intramolecular SF of pentacene dimers using time-resolved electron paramagnetic resonance measurements, TT dissociation motion was observed, and the pentacene dimer exhibited a dynamic change in the dihedral angle between chromophores.^43^ Similarly, Pc-BP-Pc is highly likely to undergo a conformational change associated with the dihedral angle change around the biphenyl linker during the TT dissociation process even under hydrostatic pressure. This was the case with the D–A–D triad that showed the excited-state conformational change under hydrostatic pressure.^38^ Hence, the nature of 
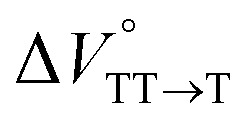
 may be attributed to the solvent reorientation/interaction occurring after the conformational change. Because the TT dissociation process is spatially divided by spin interactions, in contrast to the TT formation (*via* the exciton coupling of S_0_ and S_1_), a possible interpretation of this result is that the dependence of the solvation term (toluene *vs.* MCH) may be extremely small. The contribution of the conformational change during dissociation is further related to the stepwise reduction of *Φ*_T_ with increasing hydrostatic pressure (Table 2), which is responsible for the gradually enlarged solvent interactions in Pc-BP-Pc due to the increase in hydrostatic-pressure-driven solvent viscosity. This fact strongly supports the relationship among thermodynamically expanded or “bulkier” structures, positive 
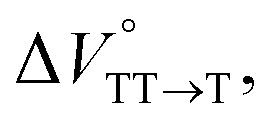
 and the gradual*τ*_T_ shortening trend. In detail, the thermodynamically bulkier T_1_ exciton by clustering a large number of solvent molecules is strongly deactivated by the solvent attack in the solvent core to shorten *τ*_T_.

**Table 2:** Singlet, correlated triplet pair, triplet quantum yields (*Φ*_S_, *Φ*_TT_, and *Φ*_T_) and reaction volume changes (Δ*V*°) of Pc-BP-Pc in toluene and MCH under hydrostatic pressure^a^

Pressure/MPa	Toluene			MCH		
	*Φ*_S_	*Φ*_TT_ b	*Φ*_T_ c	*Φ*_S_	*Φ*_TT_ b	*Φ*_T_ c
0.1	0.014	0.955	1.762	0.051	0.950	1.120
60	0.013	0.958	1.671	0.050	0.951	1.055
120	0.013	0.961	1.398	0.050	0.951	0.912
180	0.013	0.962	1.153	0.051	0.947	0.717
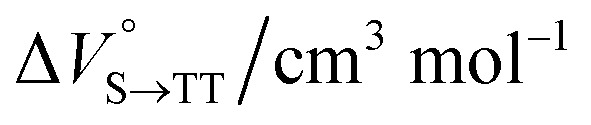	−0.8 ± 0.3			−0.1 ± 0.2		
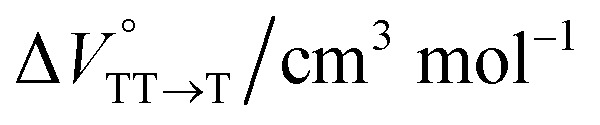	+5.9 ± 0.4			+5.8 ± 0.5		

a Measured at 298 K.

b Maximum *Φ*_TT_ = 1.

c Maximum *Φ*_T_ = 2.

## Conclusions

In this study, for the first time, we realized the significance of hydrostatic-pressure-induced intramolecular SF behavior. This was demonstrated using the biphenyl-linked pentacene dimer as a model SF material, in which hydrostatic-pressurization-based solvent-induced property changes are key factors. Although remarkable conformational changes in the dimer were not observed, through hydrostatic pressure steady-state spectrometry, the fluorescence and triplet absorption lifetime measurements enabled us to recognize that the rates of the correlated triplet pairs from the singlet were greatly accelerated by changing the hydrostatic pressure in toluene only. This revealed that the desolvation process in polar solvents plays an important role in the SF dynamics. More importantly, we have shown that the entire process of SF involving fission (S_0_ + S_1_ → TT) and dissociation (TT → 2T_1_) under hydrostatic pressure can be precisely controlled by not only kinetics in the transition state but also thermodynamics in the equilibria on the basis of microenvironmental desolvation and solvent reorientation. Finally, it should be emphasized that by using hydrostatic pressure as an external stimulus, the dynamic control concept of intramolecular SF kinetics observed in this study can be further expanded to other SF scaffolds and relevant systems that are difficult to control in both ground and excited states.

## Materials and methods

### Materials

All commercial reagents and solvents were used without further purification. Sample solutions dissolved in spectroscopic grade toluene, methylcyclohexane (MCH), and tetrahydrofuran (THF) were deaerated by five freeze–pump–thaw cycles saturated with N_2_ for fluorescence lifetime measurements or by Ar bubbling for nanosecond transient absorption (nsTA) measurements. The SF-based material (Pc-BP-Pc) was synthesized according to the literature.^17^Pc-ref was commercially available.

### Instruments

UV/vis and fluorescence spectra were recorded in a high-pressure cell (path length: 2 mm) by using a JASCO V-650 or a JASCO FP-8500. Fluorescence lifetimes were measured in a high-pressure cell by a Hamamatsu Quantaurus-Tau single photon counting apparatus fitted with an LED light source (*λ*_ex_ = 405 nm). Nanosecond transient absorption (nsTA) measurements were performed by using a Unisoku TSP-2000 flash spectrometer-pump pulse source: Surelite-I Nd:YAG (Continuum, 4–6 ns fwhm) laser with the second harmonic at 532 nm, monitor light source: xenon lamp (150 W), and detector: photomultiplier tube. ^1^H NMR spectrum of Pc-BP-Pc was recorded on an ECS-400 spectrometer.

### Hydrostatic pressure spectroscopy

Steady-state UV/vis absorption/excitation fluorescence spectroscopy and fluorescence lifetime decay measurement were conducted using a custom-built high-pressure apparatus.^28,45^ As the process has previously reported in detail in a previous study, here, we briefly describe this. A quartz inner cell was filled with the sample solution, and then the cell was set into the outer cell, where sapphire or quartz windows were fitted. A tightly closed outer cell, which was hydrostatically pressurized by water, was placed in the spectroscopic apparatus (Fig. S1a–e†).

### Hydrostatic pressure nsTA

The outer cell was placed on the nsTA optical system, which allowed us to apply hydrostatic pressure in an appropriate manner (Fig. S1f†). The T_1_ quantum yield (*Φ*_T_) of a toluene solution of Pc-BP-Pc measured in this optical system under an atmospheric pressure (0.1 MPa) was 176%, which is identical to that (*Φ*_T_ = 176%)^17^ of a THF solution observed in a regular cuvette at 0.1 MPa. This proves the validity of the nsTA measurements under hydrostatic pressure using the new optical system prepared in this study.

### Determination of S_1_ quantum yield (*Φ*_S_) of Pc-BP-Pc

Absolute S_1_ quantum yields *Φ*_P0_ (*Φ*_S_ at 0.1 MPa; 0.0140 in toluene, 0.0514 in MCH) were evaluated using a spectrofluorometer (FP-8500) fitted with an integrating sphere at 0.1 MPa, and then relative S_1_ quantum yields can be determined using the following eqn (5):^46^5
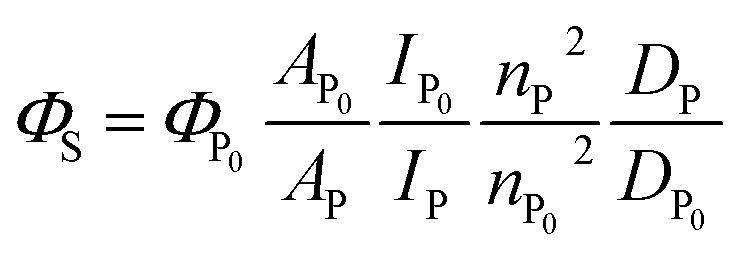
where *A*, *I*, *n*, and *D* are the absorbance at the excitation wavelength, intensity of the excitation light, refractive index, and area ratio in the fluorescence spectrum, respectively (Tables S7 and S8†). The data for *A* were extracted from Fig. 2a and S5a.† The data for *I*_P_/*I*_P_0__ were 1 because the measurements were performed at the same excitation wavelength (Fig. 2c and S6a†). The refractive indices used under atmospheric pressure (*n*_P_0__) were identical to the values reported in a previous study,^47^ and the refractive indices under pressure (*n*_P_) were calculated using the Eykman equation (eqn (6)) from the change in the density of each solvent with respect to pressure:^48,49^6
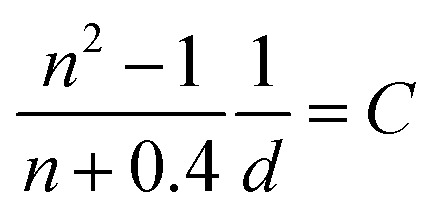


Constant *C* was calculated using a known value.^47^ The data for *D* were obtained by fitting the area of the fluorescence spectra in toluene (Fig. 2c) and MCH (Fig. S6a†) using the MATLAB software with a three-component Gaussian function (eqn (7)) (see Fig. S20 and S21 and Tables S9 and S10†):7
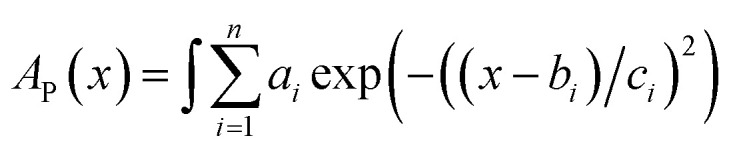


### Determination of the TT quantum yield (*Φ*_TT_) of Pc-BP-Pc

The *Φ*_TT_ values were calculated using eqn (8) (see Table S11†):8
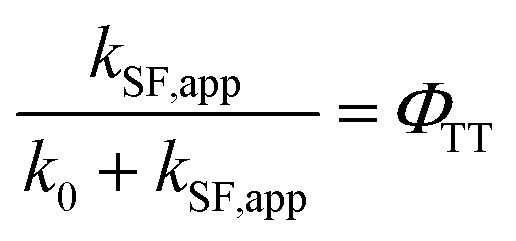
where the data for *k*_0_ and *k*_SF,app_ were extracted from Tables S2, S3 and S5,† respectively.

### Determination of T_1_ quantum yield (*Φ*_T_) of Pc-BP-Pc

The *Φ*_T,Pc-BP-Pc_ value was calculated as the relative *Φ*_T_ quantum yield (see Tables S12 and S13†) in relation to that of zinc tetraphenylporphyrin (ZnTPP) or zinc tetratolylporphyrin (ZnTTP), using the following eqn (9):^17^9
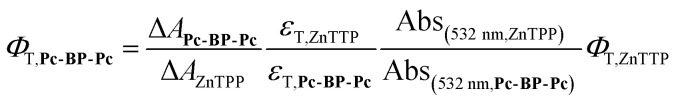
where *Φ*_T_, *ε*_T_, Δ*A* and Abs are the T_1_ quantum yield, excitation coefficient, delta absorbance in nsTA, and absorbance at 532 nm in the steady-state absorption measurements, respectively. *Φ*_T,ZnTTP_ and *ε*_T,ZnTTP_ were acquired from a previous study^50^ (*λ*_ex_ = 532 nm, *λ*_obs_ = 470 nm in toluene; *Φ*_T,ZnTTP_ = 0.88, *ε*_T,ZnTTP_ = 69 000 M^−1^ cm^−1^), and *ε*_T,Pc-BP-Pc_ from another paper^17^ (*λ*_ex_ = 532 nm, *λ*_obs_ = 516 nm in THF; *ε*_T,ZnTPP_ = 54 900 M^−1^ cm^−1^). Δ*A*_ZnTPP_ was observed at 470 nm and Δ*A*_Pc-BP-Pc_ was observed at 521 nm for toluene and 516 nm for MCH, taking into account the solvent-induced shift of the excited-state absorption T_1_–T_*n*_ band (Fig. 4a). Data of Abs_(532 nm,ZnTPP)_ and Abs_(532 nm,__Pc-BP-Pc__)_ are shown in Fig. S23b, 2a, and S5a,† respectively.

## Data availability

The data supporting this article have been uploaded as part of the ESI.†

## Author contributions

G. F. initiated and supervised the whole study. G. F. and T. H. designed the project and the experiments. T. K. performed the spectroscopic experiments. S. N. prepared the materials. M. H. designed and prepared the foundation for the transient absorption spectrometry under hydrostatic pressure. All authors contributed to writing the manuscript.

## Conflicts of interest

There are no conflicts to declare.

## Supplementary Material

SC-014-D3SC00312D-s001

## References

[cit1] Smith M. B., Michl J. (2010). Chem. Rev..

[cit2] Miyata K., Conrad-Burton F. S., Geyer F. L., Zhu X.-Y. (2019). Chem. Rev..

[cit3] Ullrich T., Munz D., Guldi D. M. (2021). Chem. Soc. Rev..

[cit4] Yang Z., Mao Z., Xie Z., Zhang Y., Liu S., Zhao J., Xu J., Chi Z., Aldred M. P. (2017). Chem. Soc. Rev..

[cit5] Mei J., Leung N. L. C., Kwok R. T. K., Lam J. W. Y., Tang B. Z. (2015). Chem. Rev..

[cit6] Yanai N., Kimizuka N. (2017). Acc. Chem. Res..

[cit7] Bell T. W., Hext N. M. (2004). Chem. Soc. Rev..

[cit8] You L., Zha D., Anslyn E. V. (2015). Chem. Rev..

[cit9] Fukuhara G. (2020). J. Photochem. Photobiol., C.

[cit10] Einzinger M., Wu T., Kompalla J. F., Smith H. L., Perkinson C. F., Nienhaus L., Wieghold S., Congreve D. N., Kahn A., Bawendi M. G., Baldo M. A. (2019). Nature.

[cit11] Reusswig P. D., Congreve D. N., Thompson N. J., Baldo M. A. (2012). Appl. Phys. Lett..

[cit12] Saegusa T., Sakai H., Nagashima H., Kobori Y., Tkachenko N. V., Hasobe T. (2019). J. Am. Chem. Soc..

[cit13] Burdett J. J., Gosztola D., Bardeen C. J. (2011). J. Chem. Phys..

[cit14] Wilson M. W. B., Rao A., Johnson K., Gélinas S., di Pietro R., Clark J., Friend R. H. (2013). J. Am. Chem. Soc..

[cit15] Nakamura S., Sakai H., Fuki M., Kobori Y., Tkachenko N. V., Hasobe T. (2021). J. Phys. Chem. Lett..

[cit16] Zhao X., O'Connor J. P., Schultz J. D., Bae Y. J., Lin C., Young R. M., Wasielewski M. R. (2021). J. Phys. Chem. B.

[cit17] Sakuma T., Sakai H., Araki Y., Mori T., Wada T., Tkachenko N. V., Hasobe T. (2016). J. Phys. Chem. A.

[cit18] Alvertis A. M., Lukman S., Hele T. J. H., Fuemmeler E. G., Feng J., Wu J., Greenham N. C., Chin A. W., Musser A. J. (2019). J. Am. Chem. Soc..

[cit19] Aster A., Zinna F., Rumble C., Lacour J., Vauthey E. (2021). J. Am. Chem. Soc..

[cit20] Grieco C., Doucette G. S., Pensack R. D., Payne M. M., Rimshaw A., Scholes G. D., Anthony J. E., Asbury J. B. (2016). J. Am. Chem. Soc..

[cit21] Dvořák M., Prasad S. K. K., Dover C. B., Forest C. R., Kaleem A., MacQueen R. W., Petty II A. J., Forecast R., Beves J. E., Anthony J. E., Tayebjee M. J. Y., Widmer-Cooper A., Thordarson P., Schmidt T. W. (2021). J. Am. Chem. Soc..

[cit22] Zhang J., Sakai H., Suzuki K., Hasobe T., Tkachenko N. V., Chang I.-Y., Hyeon-Deuk K., Kaji H., Teranishi T., Sakamoto M. (2021). J. Am. Chem. Soc..

[cit23] Sakai H., Yoshino K., Shoji Y., Kajitani T., Pu J., Fukushima T., Takenobu T., Tkachenko N. V., Hasobe T. (2022). J. Phys. Chem. C.

[cit24] Tilley A. J., Pensack R. D., Kynaston E. L., Scholes G. D., Seferos D. S. (2018). Chem. Mater..

[cit25] Catti L., Narita H., Tanaka Y., Sakai H., Hasobe T., Tkachenko N. V., Yoshizawa M. (2021). J. Am. Chem. Soc..

[cit26] Drljaca A., Hubbard C. D., van Eldik R., Asano T., Basilevsky M. V., le Noble W. J. (1998). Chem. Rev..

[cit27] Silva J. L., Oliveira A. C., Vieira T. C. R. G., de Oliveira G. A. P., Suarez M. C., Foguel D. (2014). Chem. Rev..

[cit28] Mizuno H., Fukuhara G. (2022). Acc. Chem. Res..

[cit29] Bovey F. A., Yanari S. S. (1960). Nature.

[cit30] Johnson P. C., Offen H. W. (1972). J. Chem. Phys..

[cit31] Hara K., Obara K. (1985). Chem. Phys. Lett..

[cit32] Hara K., Arese T., Osugi J. (1984). J. Am. Chem. Soc..

[cit33] Hara K., Suzuki H., Rettig W. (1988). Chem. Phys. Lett..

[cit34] Lee R., Howard J. A. K., Probert M. R., Steed J. W. (2014). Chem. Soc. Rev..

[cit35] O'Bannon III E. F., Jenei Z., Cynn H., Lipp M. J., Jeffries J. R. (2018). Rev. Sci. Instrum..

[cit36] Sagara Y., Kato T. (2009). Nat. Chem..

[cit37] Krieg M., Fläschner G., Alsteens D., Gaub B. M., Roos W. H., Wuite G. J. L., Gaub H. E., Gerber C., Dufrêne Y. F., Müller D. J. (2019). Nat. Rev. Phys..

[cit38] Takeda Y., Mizuno H., Okada Y., Okazaki M., Minakata S., Penfold T., Fukuhara G. (2019). ChemPhotoChem.

[cit39] Nakasha K., Fukuhara G. (2020). ACS Appl. Polym. Mater..

[cit40] Doucette G. S., Huang H.-T., Munro J. M., Munson K. T., Park C., Anthony J. E., Strobel T., Dabo I., Badding J. V., Asbury J. B. (2020). Cell Rep. Phys. Sci..

[cit41] Nakamura S., Sakai H., Nagashima H., Fuki M., Onishi K., Khan R., Kobori Y., Tkachenko N. V., Hasobe T. (2021). J. Phys. Chem. C.

[cit42] Sakai H., Inaya R., Nagashima H., Nakamura S., Kobori Y., Tkachenko N. V., Hasobe T. (2018). J. Phys. Chem. Lett..

[cit43] Kobori Y., Fukui M., Nakamura S., Hasobe T. (2020). J. Phys. Chem. B.

[cit44] Lukman S., Chen K., Hodgkiss J. M., Turban D. H. P., Hine N. D. M., Dong S., Wu J., Greenham N. C., Musser A. J. (2016). Nat. Commun..

[cit45] Ayitou A. J.-L., Fukuhara G., Kumarasamy E., Inoue Y., Sivaguru J. (2013). Chem.–Eur. J..

[cit46] Demas J. N., Crosby G. A. (1971). J. Phys. Chem..

[cit47] González B., Domínguez I., González E. J., Domínguez Á. (2010). J. Chem. Eng. Data.

[cit48] Assael M. J., Avelino H. M. T., Dalaouti N. K., Fareleira J. M. N. A., Harris K. R. (2001). Int. J. Thermophys..

[cit49] Dakkach M., Rubio-Pérez G., Alaoui F. E. M., Muñoz-Rujas N., Aguilar F., Montero E. A. (2020). J. Chem. Eng. Data.

[cit50] Tran-Thi T. H., Desforge C., Thiec C., Gaspard S. (1989). J. Phys. Chem..

