# Supplementary material for: Control of intramolecular singlet fission in a pentacene dimer by hydrostatic pressure
Source: Chem Sci. 2023 Feb 23;14(12):3293–301. doi: 10.1039/d3sc00312d (PMC10034212; doi:10.1039/d3sc00312d)
Supplement: SC-014-D3SC00312D-s001 [file SC-014-D3SC00312D-s001.pdf]

Electronic Supplementary Material (ESI) for Chemical Science.
This journal is © The Royal Society of Chemistry 2023

Electronic Supplementary Information
for

Control of intramolecular singlet fission in a pentacene dimer by hydrostatic pressure

Tomokazu Kinoshita,[a] Shunta Nakamura,[b] Makoto Harada,[a] Taku Hasobe[*b] and Gaku Fukuhara[*a]

[a] *Department of Chemistry, Tokyo Institute of Technology, 2-12-1 Ookayama, Meguro-ku, Tokyo 152-8551, Japan*

[b] *Department of Chemistry, Faculty of Science and Technology, Keio University, Yokohama, Kanagawa 223-8522, Japan*

E-mail: hasobe@chem.keio.ac.jp (T.H.), gaku@chem.titech.ac.jp (G.F.)

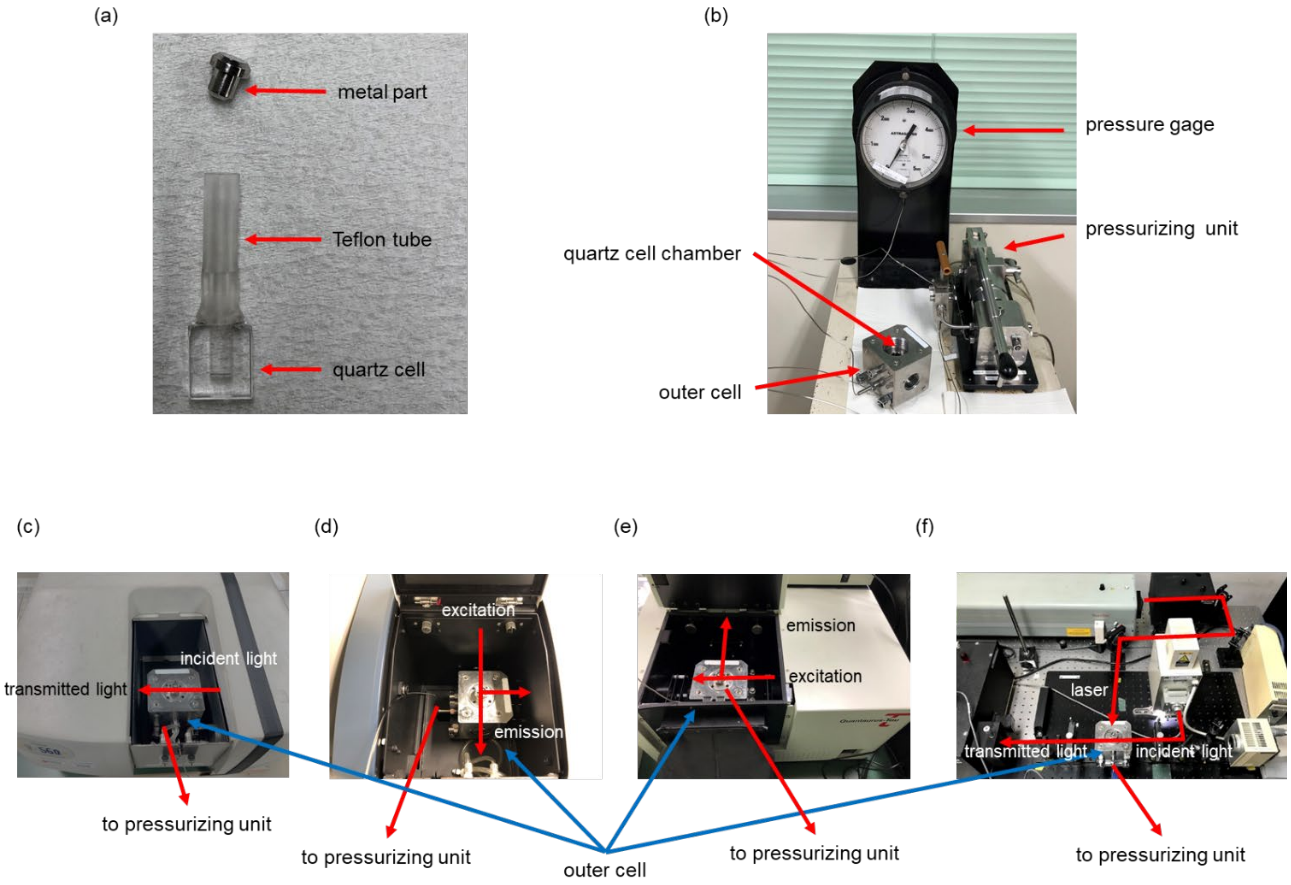

Figure S1. Photographs of the (a) inner cell, (b) high-pressurizing unit, and set up for the (c) UV/vis absorption, (d) fluorescence/excitation, (e) time-correlated single-photon counting, and (f) nanosecond transient absorption (nsTA) measurements.

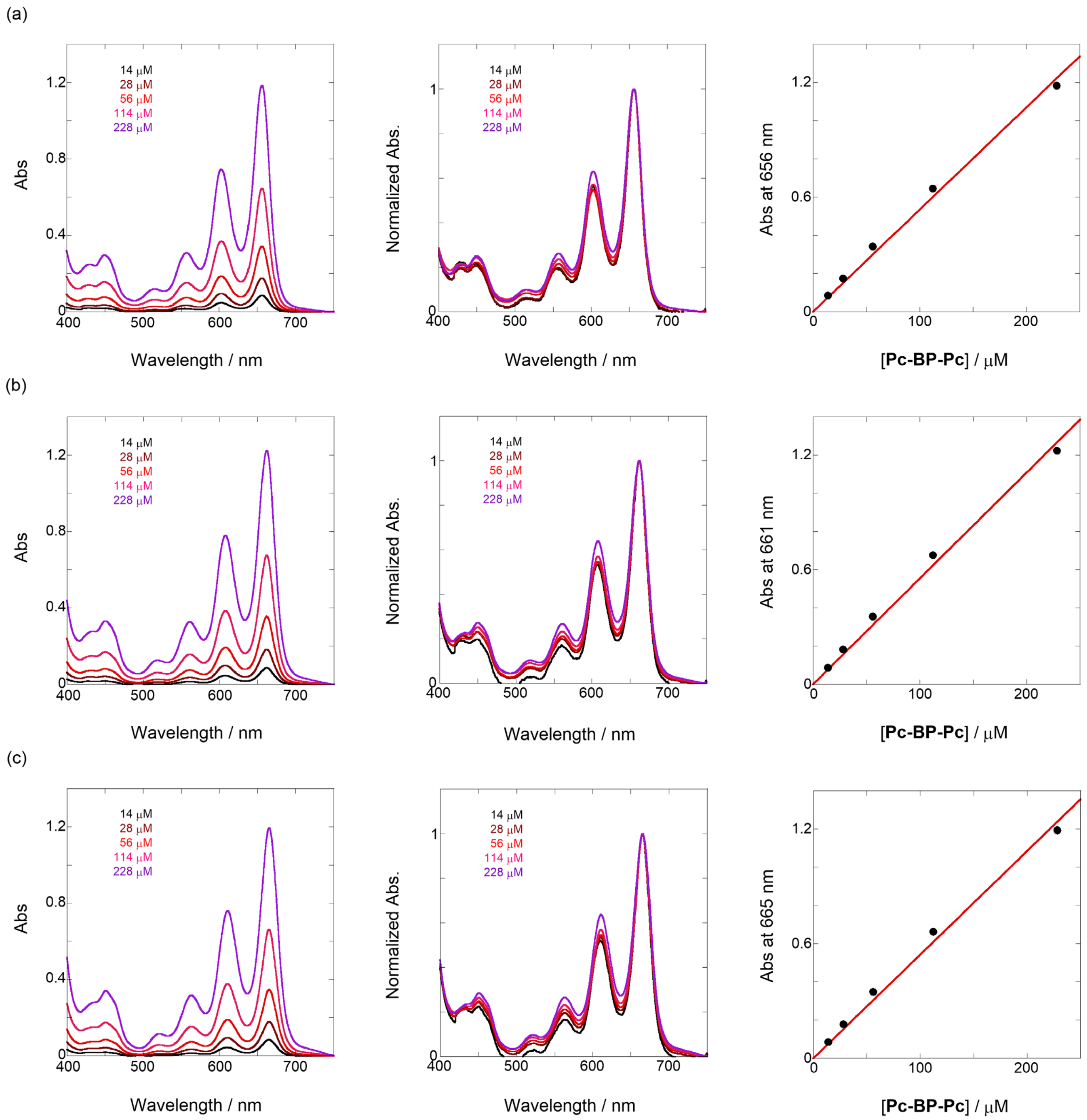

Figure S2. Concentration-dependent (14-228 μM, from black to purple) UV/vis spectra (*left*), normalized UV/vis spectra (*center*), and absorbance plots at the 0-0 absorption maxima (*right*) of **Pc-BP-Pc** at (a) 0.1 MPa (correlation coefficient $r = 0.996$), (b) 160 MPa ($r = 0.996$), and (c) 320 MPa ($r = 0.995$) in toluene at room temperature, measured in a high-pressure cell.

Table S1. Hydrostatic Pressure-Induced Spectral Shifts of Absorption and Fluorescence Maxima

Solvent	Dipole moment / D	Slopes of the absorption 0–0 band/$cm^{-1}MPa^{-1}$		Slopes of the fluorescence 0–0 band/$cm^{-1}MPa^{-1}$	
		**Pc-BP-Pc**	**Pc-ref**	**Pc-BP-Pc**	**Pc-ref**
MCH	0.00	-0.660	-0.727	-0.588	-0.755
Toluene	0.38	-0.750	-0.848	-0.856	-0.891
THF	1.75	-0.636	*a*	-0.729	*a*

[a]Not determined.

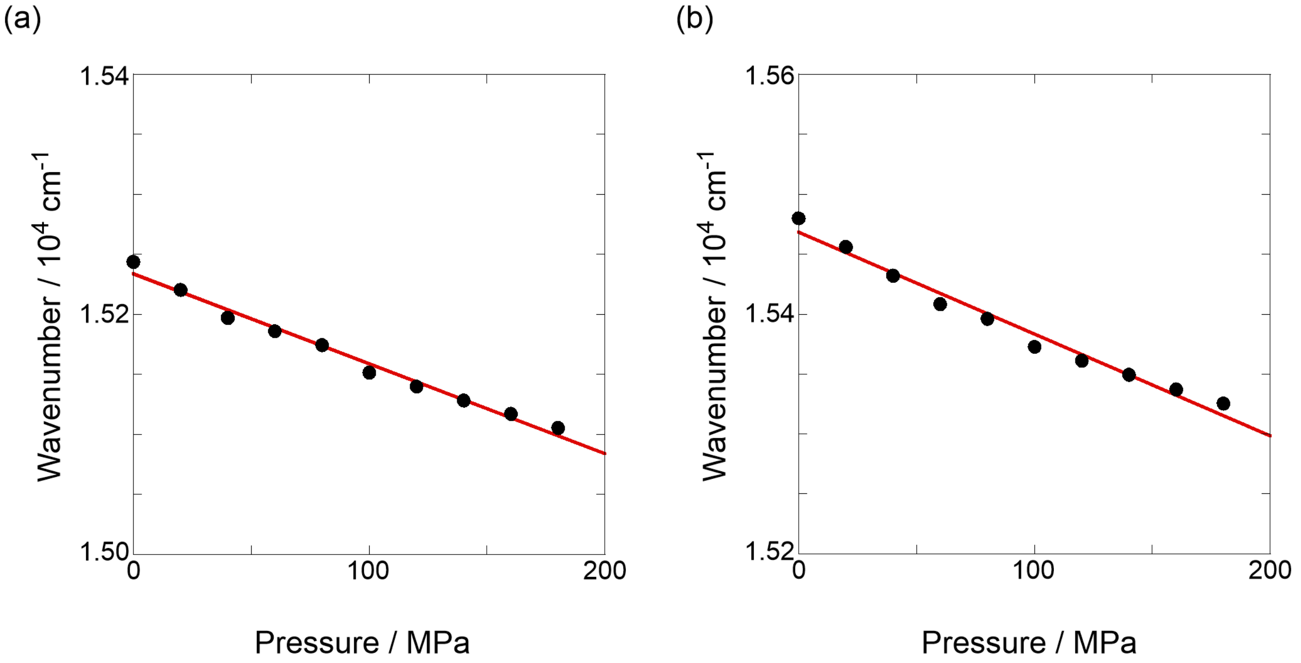

Figure S3. Plots of wavenumber of the absorption maxima at the 0–0 band of (a) **Pc-BP-Pc** (82 μM) (r = 0.993, slope = -0.750 cm^{-1} MPa^{-1}) and (b) **Pc-ref** (91 μM) (r = 0.989, slope = -0.848 cm^{-1} MPa^{-1}) in toluene at room temperature at 0.1, 20, 40, 60, 80, 100, 120, 140, 160, and 180 MPa, measured in a high-pressure cell.

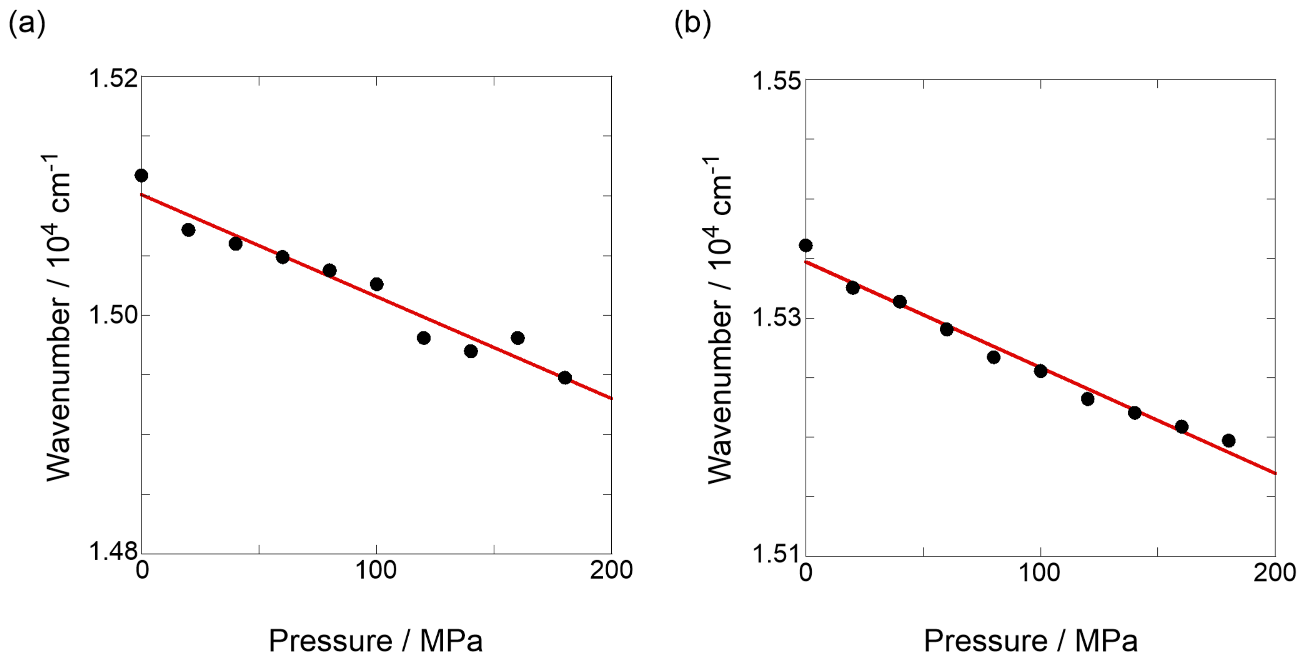

Figure S4. Plots of wavenumber of the fluorescence maxima at the 0–0 band of (a) **Pc-BP-Pc** (67 μM) (λ_{ex} = 580 nm, r = 0.974, slope = -0.856 cm^{-1} MPa^{-1}) and (b) **Pc-ref** (91 μM) (λ_{ex} = 543 nm, r = 0.990, slope = -0.891 cm^{-1} MPa^{-1}) in toluene at room temperature at 0.1, 20, 40, 60, 80, 100, 120, 140, 160, and 180 MPa, measured in a high-pressure cell.

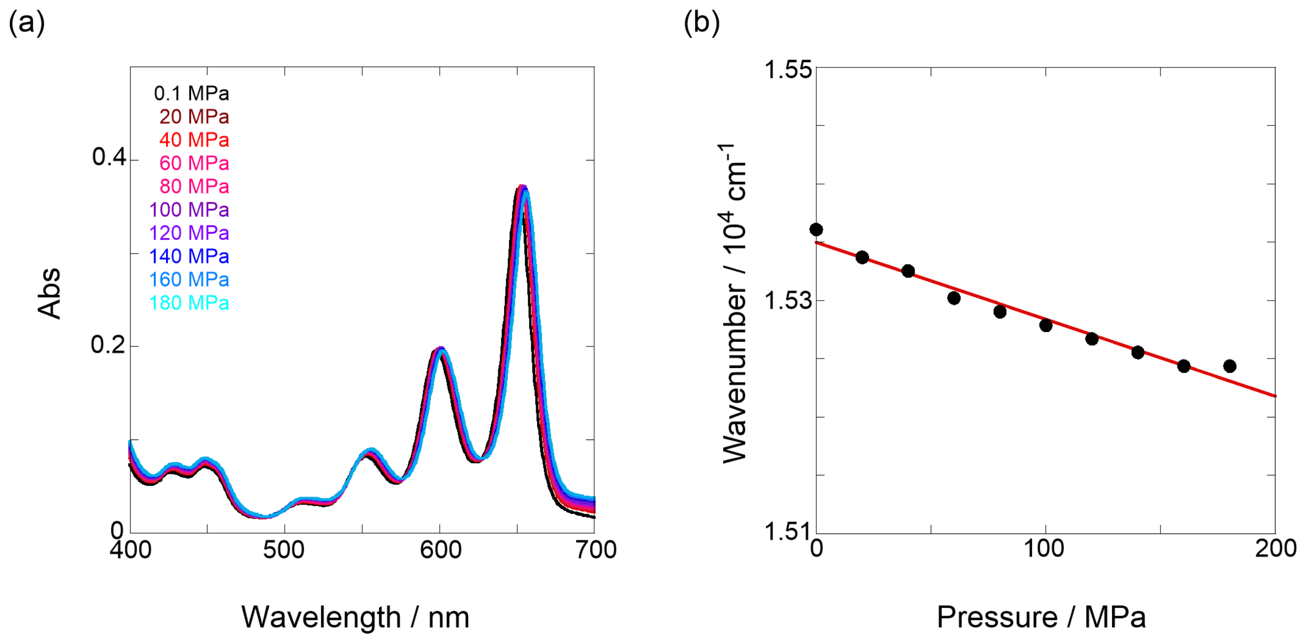

Figure S5. (a) Pressure-dependent UV/vis spectra and (b) plot of wavenumber of the absorption maxima at the 0–0 band of **Pc-BP-Pc** (91 μM) (r = 0.985, slope = -0.660 cm^{-1} MPa^{-1}) in MCH at room temperature at 0.1, 20, 40, 60, 80, 100, 120, 140, 160, and 180 MPa (from black to sky blue), measured in a high-pressure cell.

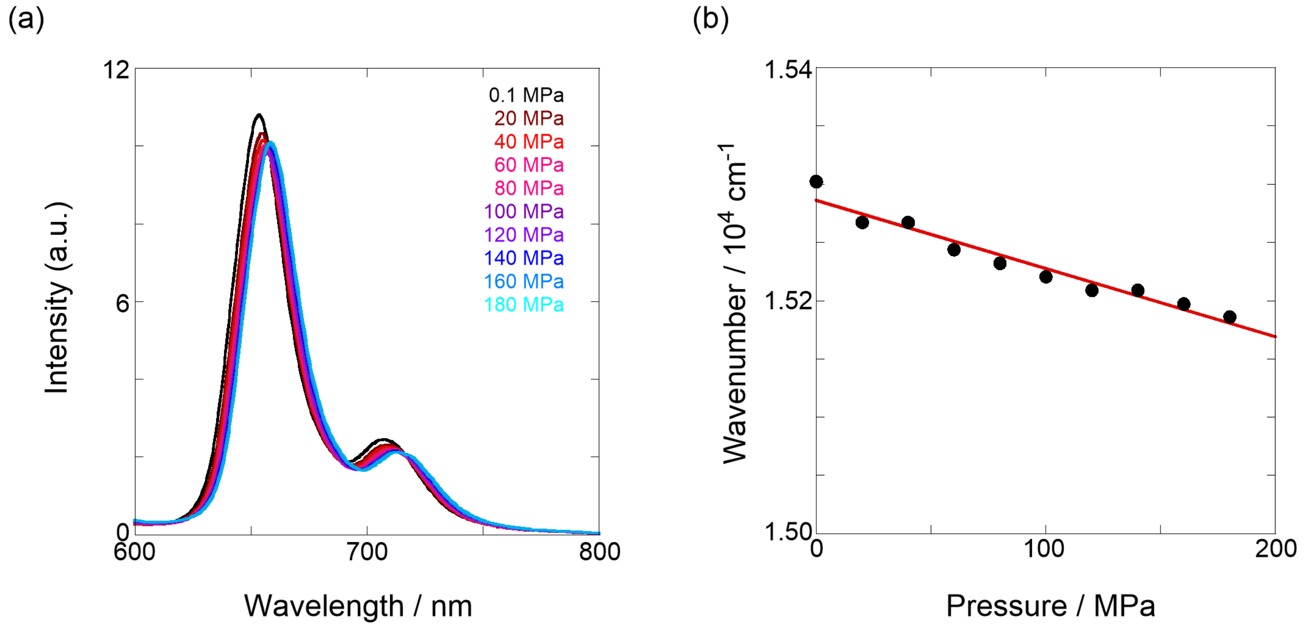

Figure S6. (a) Pressure-dependent fluorescence spectra and (b) plot of wavenumber of the fluorescence maxima at the 0–0 band of **Pc-BP-Pc** (87 μM) (λ_{ex} = 576 nm, r = 0.975, slope = -0.588 cm^{-1} MPa^{-1}) in MCH at room temperature at 0.1, 20, 40, 60, 80, 100, 120, 140, 160, and 180 MPa (from black to sky blue), measured in a high-pressure cell.

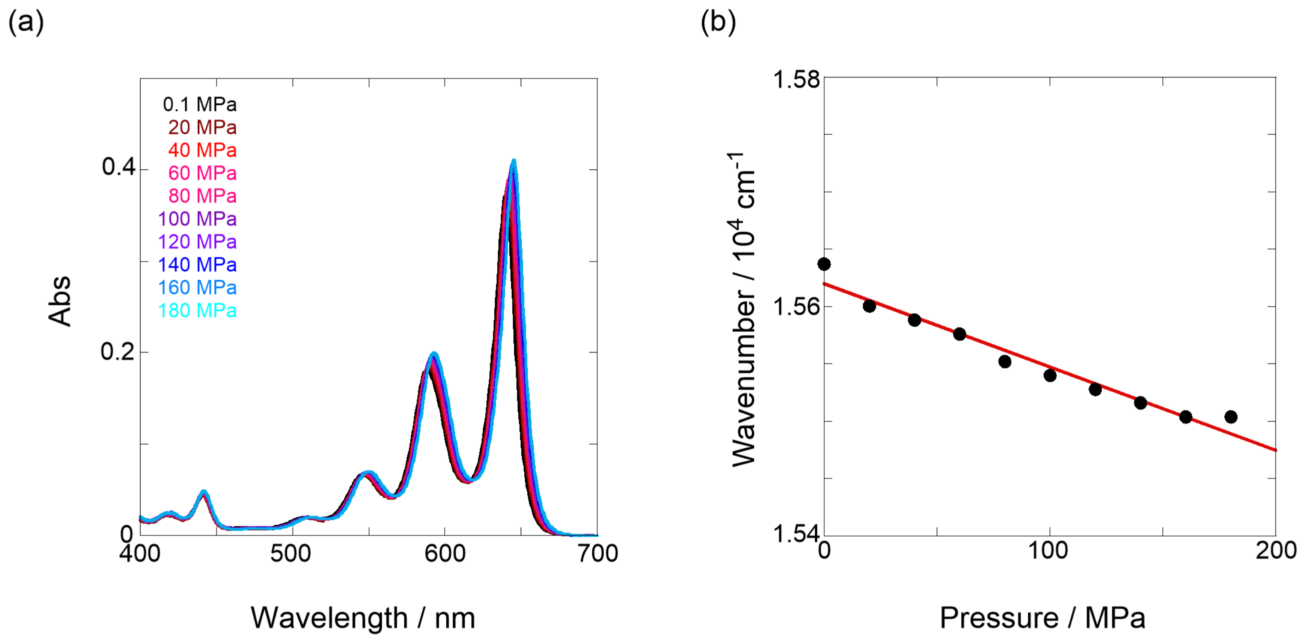

Figure S7. (a) Pressure-dependent UV/vis spectra and (b) plot of wavenumber of the absorption maxima at the 0–0 band of **Pc-ref** (111 μM) (r = 0.980, slope = -0.727 cm^{-1} MPa^{-1}) in MCH at room temperature at 0.1, 20, 40, 60, 80, 100, 120, 140, 160, and 180 MPa (from black to sky blue), measured in a high-pressure cell.

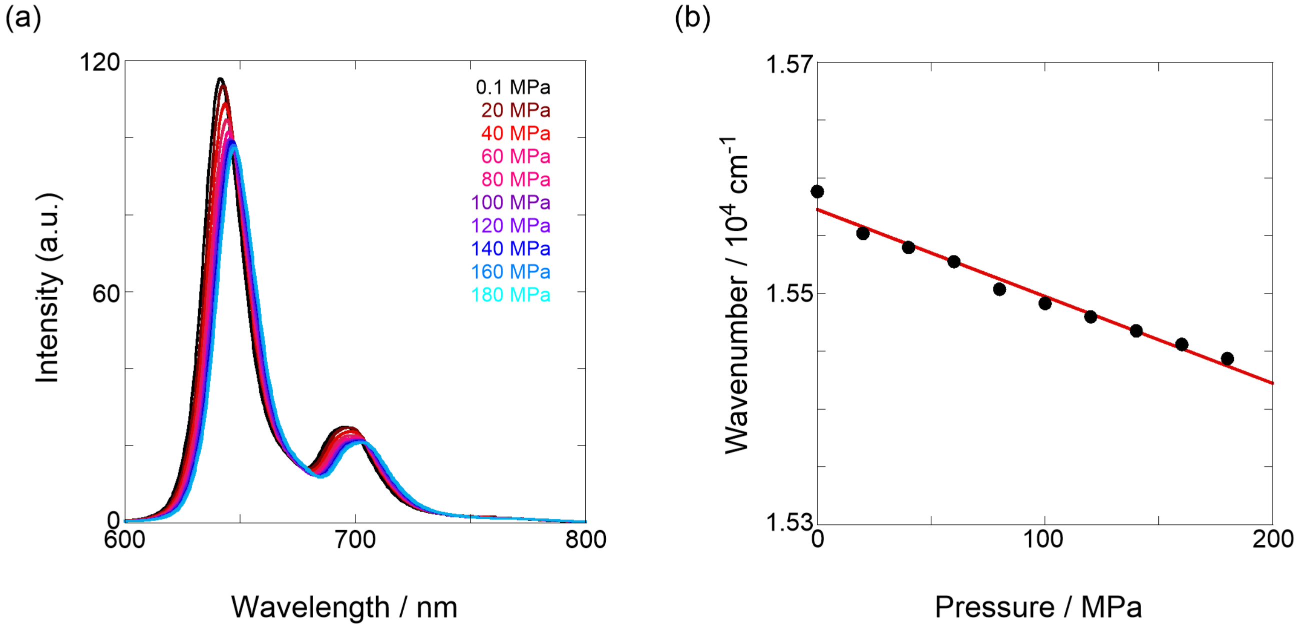

Figure S8. (a) Pressure-dependent fluorescence spectra and (b) plot of wavenumber of the fluorescence maxima at the 0–0 band of **Pc-ref** (111 μM) (λ_{ex} = 568 nm, r = 0.988, slope = -0.755 cm^{-1} MPa^{-1}) in MCH at room temperature at 0.1, 20, 40, 60, 80, 100, 120, 140, 160, and 180 MPa (from black to sky blue), measured in a high-pressure cell.

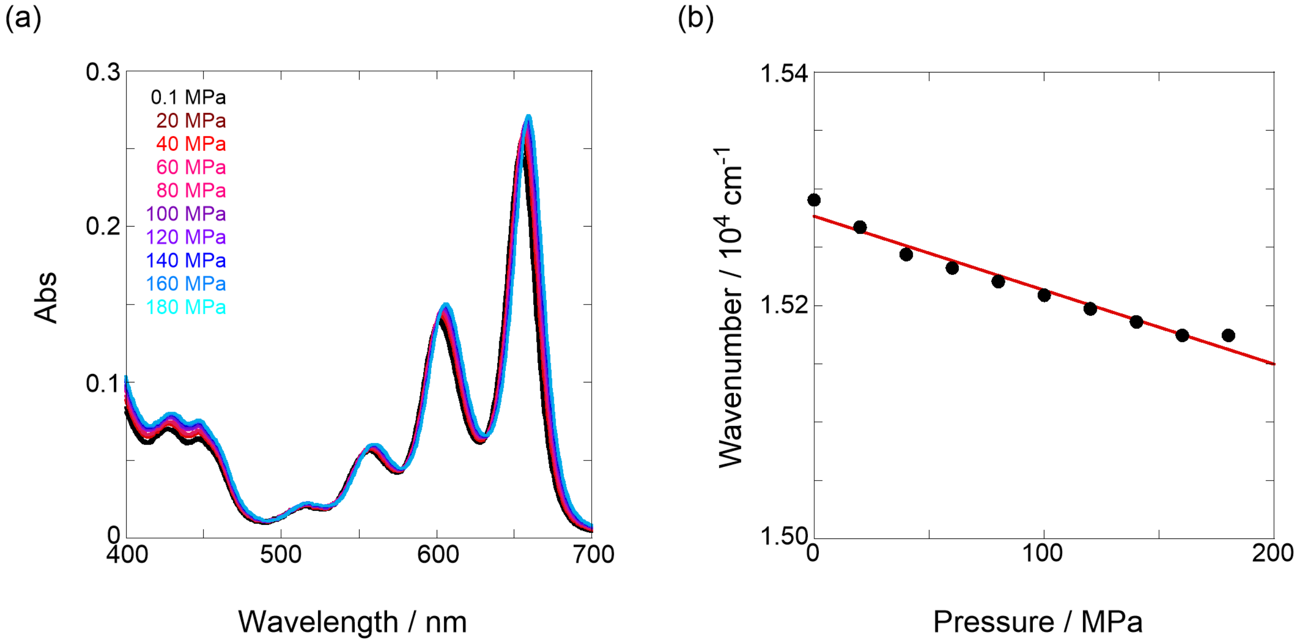

Figure S9. (a) Pressure-dependent UV/vis spectra and (b) plot of wavenumber of the absorption maxima at the 0–0 band of **Pc-BP-Pc** (77 μM) (r = 0.982, slope = -0.636 cm^{-1} MPa^{-1}) in THF at room temperature at 0.1, 20, 40, 60, 80, 100, 120, 140, 160, and 180 MPa (from black to sky blue), measured in a high-pressure cell.

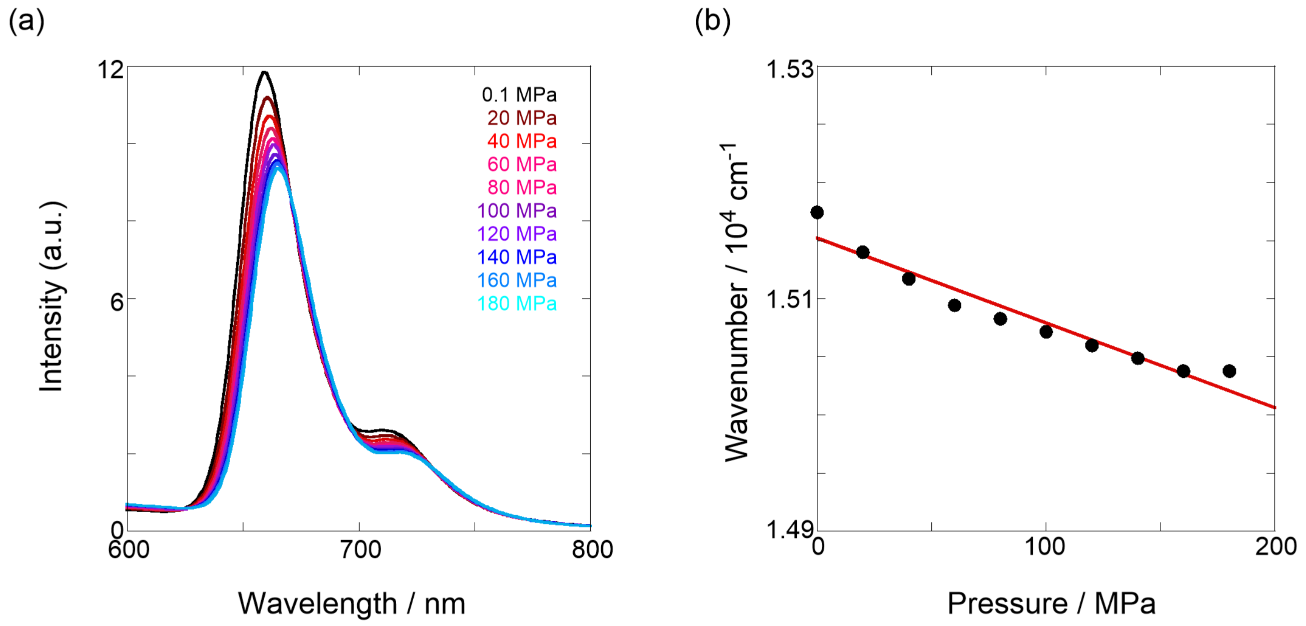

Figure S10. (a) Pressure-dependent fluorescence spectra and (b) plot of wavenumber of the fluorescence maxima at the 0–0 band of **Pc-BP-Pc** (77 μM) (λ_{ex} = 580 nm, r = 0.967, slope = -0.729 cm^{-1} MPa^{-1}) in THF at room temperature at 0.1, 20, 40, 60, 80, 100, 120, 140, 160, and 180 MPa (from black to sky blue), measured in a high-pressure cell.

Table S2. Fluorescence Lifetimes of Pc-BP-Pc and Pc-ref in Toluene under Hydrostatic Pressure[a]

Sample	λ_{em}[b]/nm	Pressure/MPa	τ_1/ns	τ_1 / %	τ_2/ns	τ_2 / %	χ^2
**Pc-BP-Pc** (74 μM)	660	0.1	14.8	0.70	0.6	0.30	1.1
		20	14.6	0.72	0.7	0.28	1.2
		40	14.6	0.67	0.6	0.33	1.3
		60	14.5	0.66	0.5	0.34	1.1
		80	14.4	0.65	0.5	0.35	1.1
		100	14.3	0.65	0.6	0.35	1.2
		120	14.3	0.62	0.5	0.38	1.2
		140	14.3	0.61	0.5	0.39	1.2
		160	14.1	0.61	0.6	0.39	1.2
		180	14.0	0.59	0.5	0.41	1.2
**Pc-ref** (89 μM)	650	0.1	12.9	1.00			1.1
		20	12.8	1.00			1.2
		40	12.7	1.00			1.2
		60	12.7	1.00			1.3
		80	12.7	1.00			1.2
		100	12.8	1.00			1.2
		120	12.7	1.00			1.1
		140	12.8	1.00			1.1
		160	12.7	1.00			1.1
		180	12.7	1.00			1.1

[a]Fluorescence lifetime (τ_i) and population (%) of each component, determined by the hydrostatic pressure single photon counting method in degassed solution at room temperature; λ_{ex} = 405 nm. [b]Monitoring wavelength.

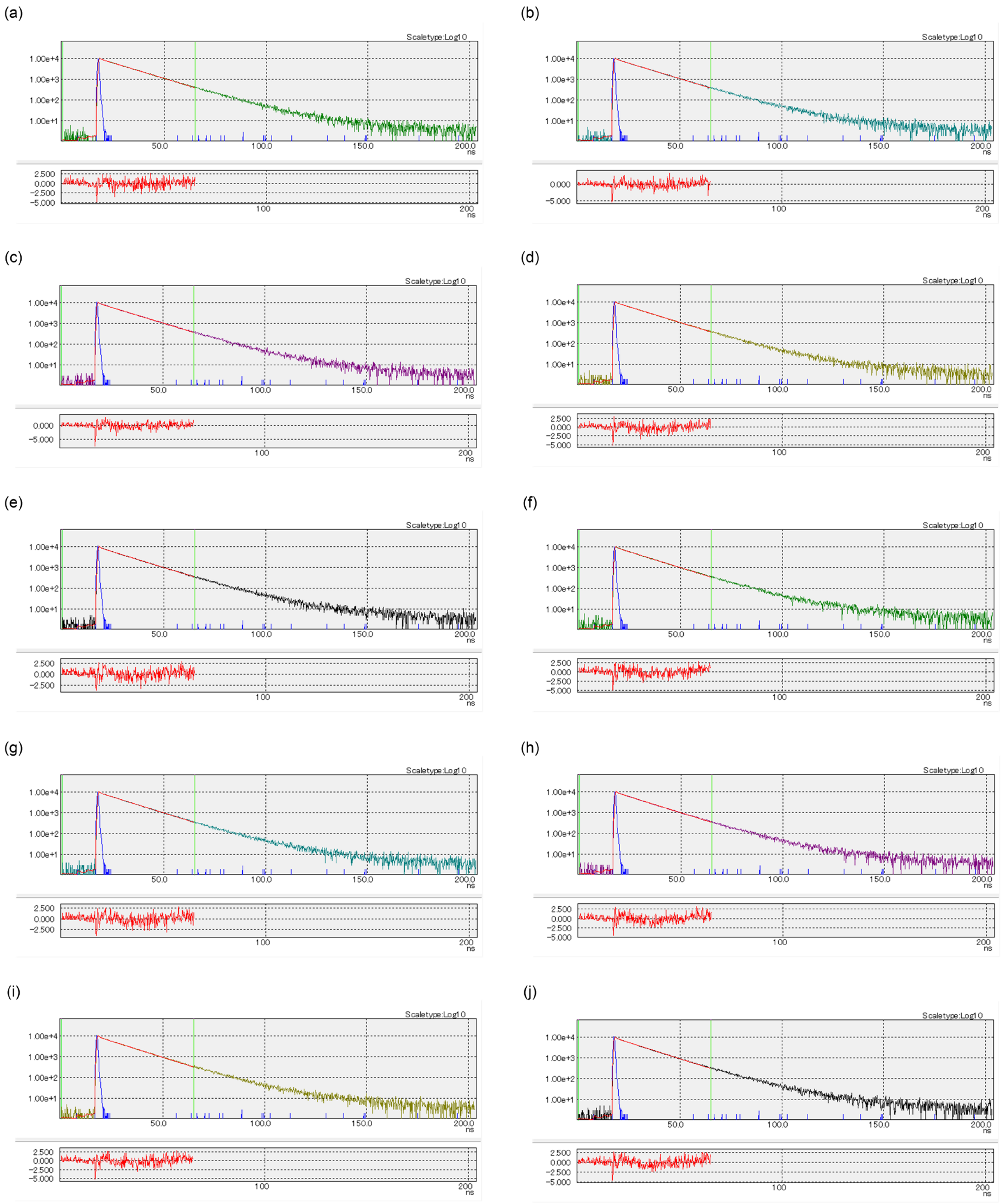

Figure S11. Time-correlated fluorescence decays of **Pc-BP-Pc** (74 μM) monitored at 660 nm at (a) 0.1, (b) 20, (c) 40, (d) 60, (e) 80, (f) 100, (g) 120, (h) 140, (i) 160, and (j) 180 MPa in toluene at room temperature, measured in a high-pressure cell, where the colored, red, and blue lines represent the fluorescence decay, fitting result, and the instrument response function, respectively.

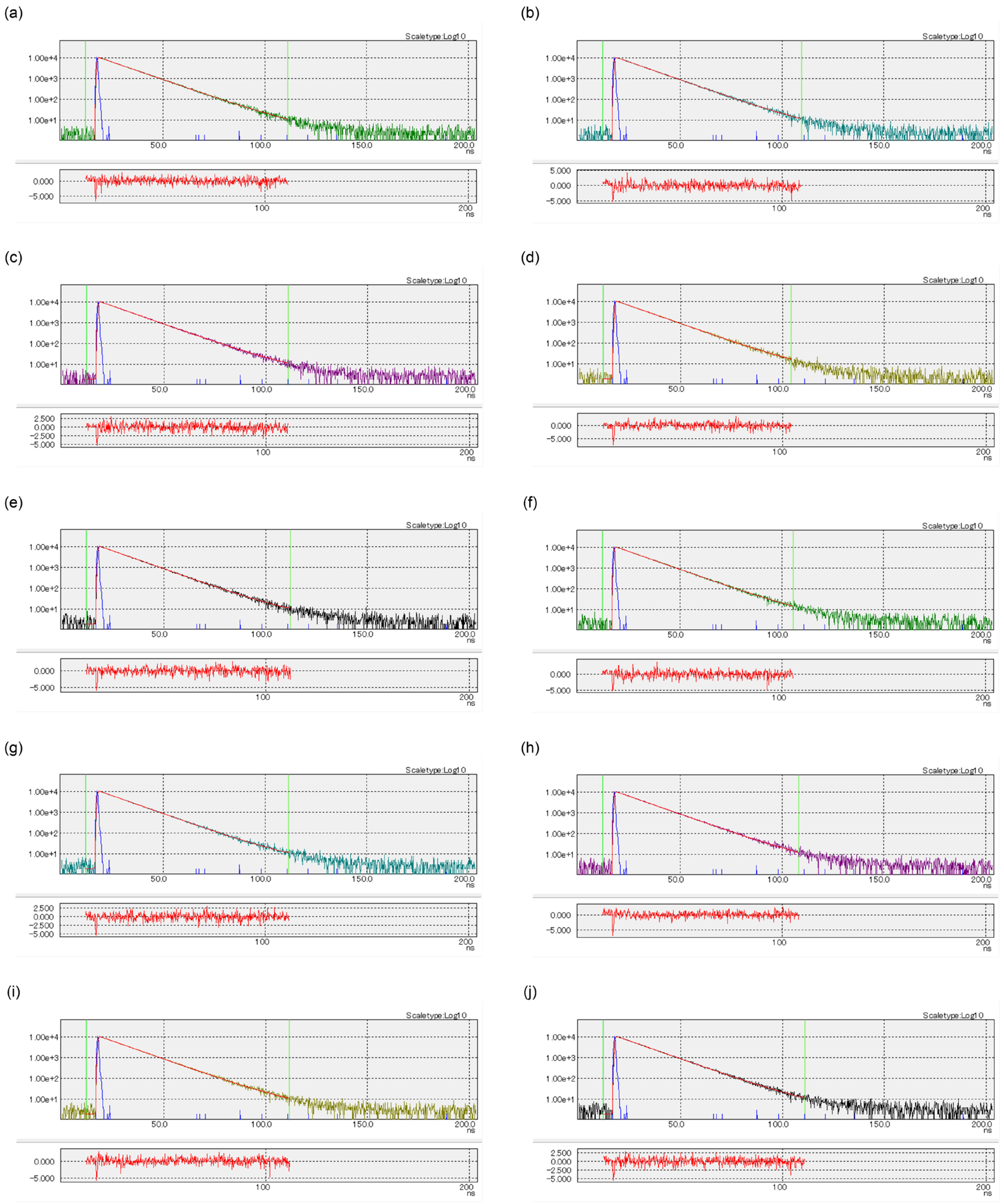

Figure S12. Time-correlated fluorescence decays of **Pc-ref** (89 μM) monitored at 650 nm at (a) 0.1, (b) 20, (c) 40, (d) 60, (e) 80, (f) 100, (g) 120, (h) 140, (i) 160, and (j) 180 MPa in toluene at room temperature, measured in a high-pressure cell, where the colored, red, and blue lines represent the fluorescence decay, fitting result, and the instrument response function, respectively.

Table S3. Fluorescence Lifetimes of Pc-BP-Pc and Pc-ref in MCH under Hydrostatic Pressure[a]

Sample	$\lambda_{em}{}^{b}$/nm	Pressure/MPa	τ_1/ns	τ_1 / %	τ_2/ns	τ_2 / %	χ^2
**Pc-BP-Pc** (88 μM)	653	0.1	14.9	0.83	0.8	0.17	1.1
		20	14.9	0.83	0.8	0.17	1.2
		40	15.0	0.81	0.8	0.19	1.2
		60	15.2	0.80	0.7	0.20	1.2
		80	15.3	0.78	0.7	0.22	1.1
		100	15.5	0.78	0.8	0.22	1.1
		120	15.6	0.78	0.8	0.22	1.2
		140	15.8	0.77	0.7	0.23	1.1
		160	15.9	0.79	0.8	0.21	1.2
		180	16.0	0.79	0.8	0.21	1.2
**Pc-ref** (111 μM)	642	0.1	15.2	1.00			1.3
		20	15.0	1.00			1.0
		40	15.0	1.00			1.2
		60	15.0	1.00			1.1
		80	15.1	1.00			1.1
		100	15.2	1.00			1.3
		120	15.4	1.00			1.1
		140	15.5	1.00			1.2
		160	15.6	1.00			1.2
		180	15.7	1.00			1.0

[a]Fluorescence lifetime (τ_i) and population (%) of each component, determined by the hydrostatic pressure single photon counting method in degassed solution at room temperature; λ_{ex} = 405 nm. [b]Monitoring wavelength.

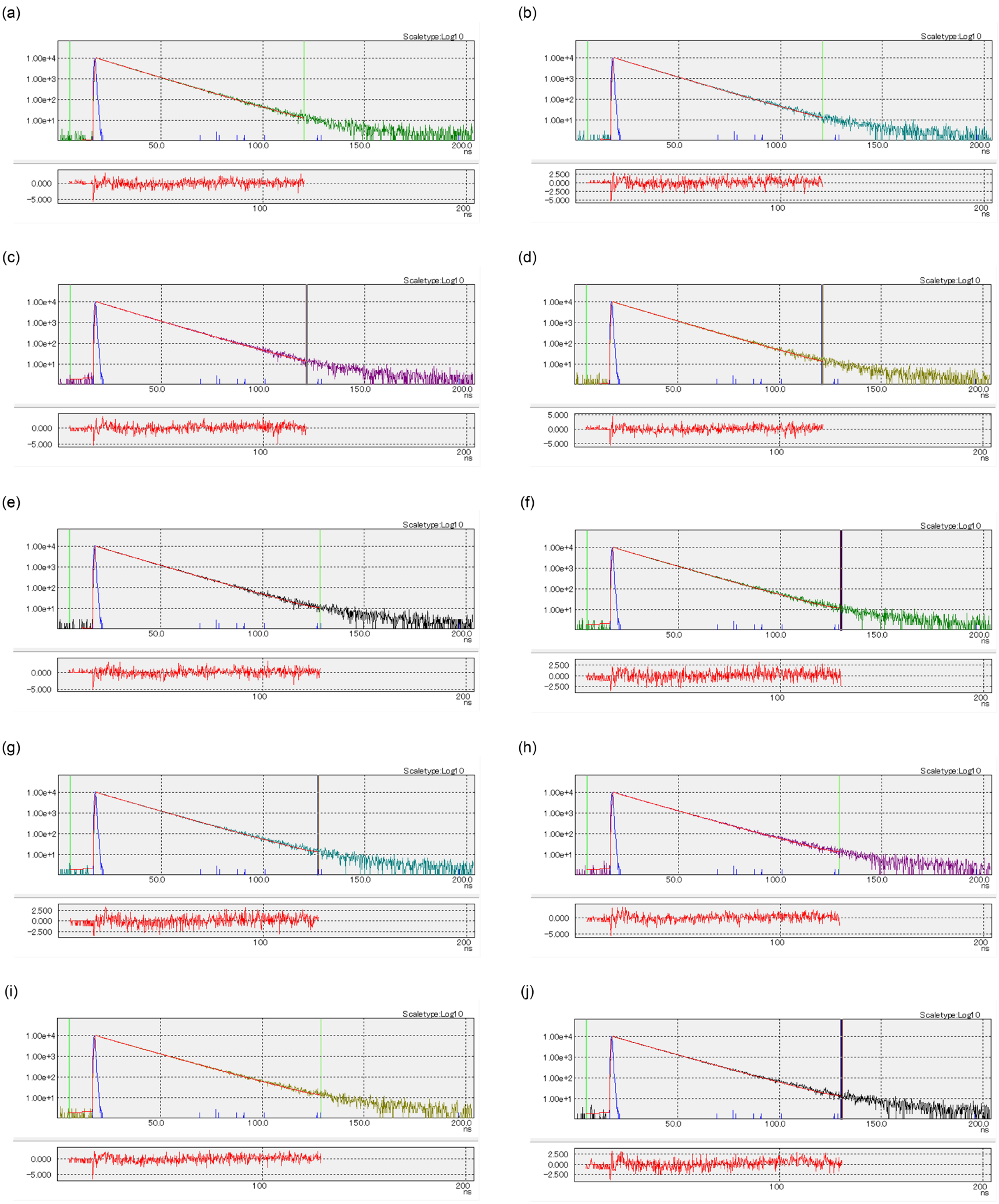

Figure S13. Time-correlated fluorescence decays of **Pc-BP-Pc** (88 μM) monitored at 653 nm at (a) 0.1, (b) 20, (c) 40, (d) 60, (e) 80, (f) 100, (g) 120, (h) 140, (i) 160, and (j) 180 MPa in MCH at room temperature, measured in a high-pressure cell, where the colored, red, and blue lines represent the fluorescence decay, fitting result, and the instrument response function, respectively.

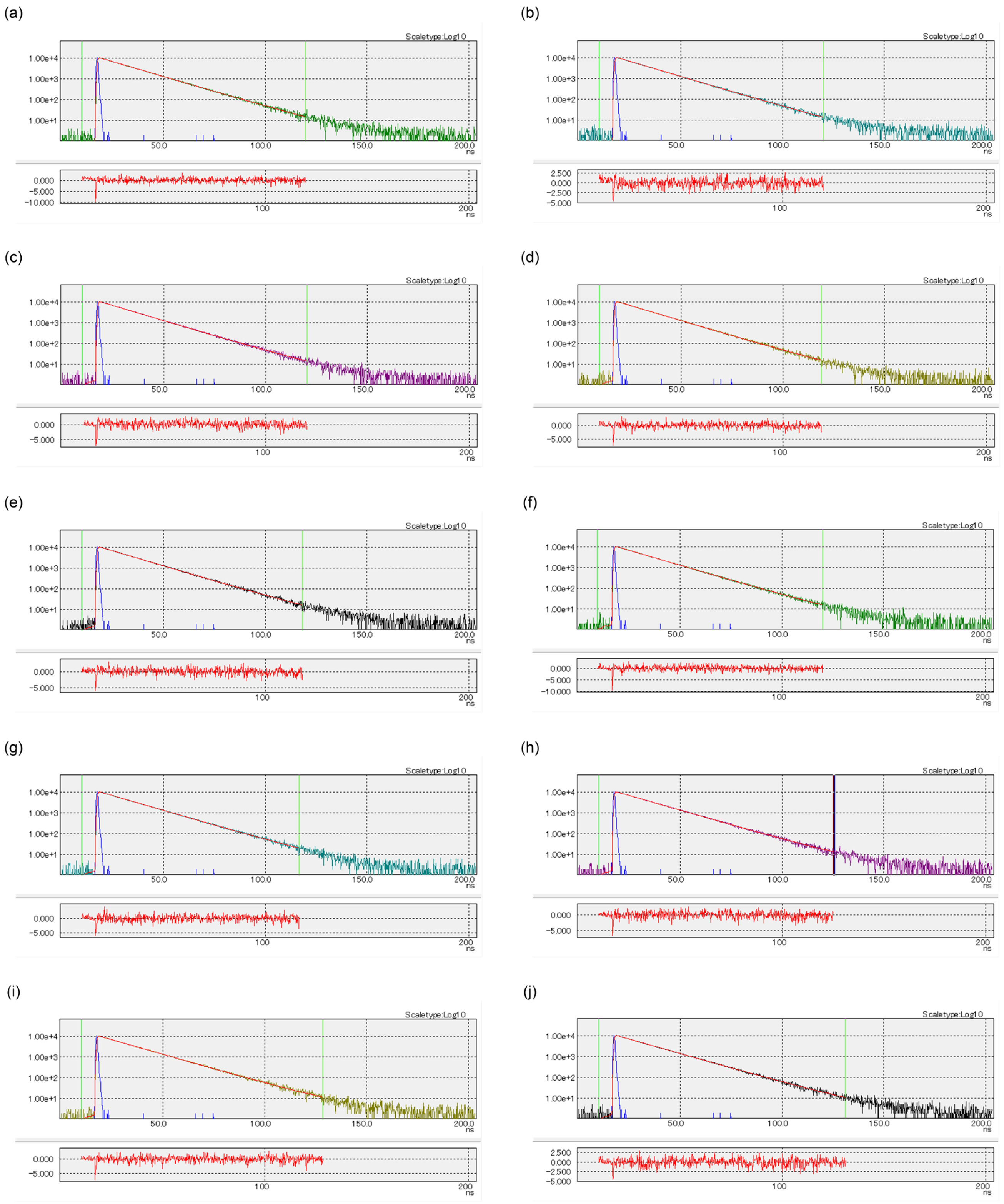

Figure S14. Time-correlated fluorescence decays of **Pc-ref** (111 μM) monitored at 642 nm at (a) 0.1, (b) 20, (c) 40, (d) 60, (e) 80, (f) 100, (g) 120, (h) 140, (i) 160, and (j) 180 MPa in MCH at room temperature, measured in a high-pressure cell, where the colored, red, and blue lines represent the fluorescence decay, fitting result, and the instrument response function, respectively.

Table S4. Fluorescence Lifetimes of Pc-BP-Pc and Pc-ref in THF under Hydrostatic Pressure[a]

Sample	λ_{em}[b]/nm	Pressure/MPa	τ_1/ns	τ_1 / %	τ_2/ns	τ_2 / %	χ^2
**Pc-BP-Pc** (77 μM)	655	40	12.3	0.71	0.8	0.29	1.2
		60	12.1	0.70	0.7	0.30	1.2
		80	12.0	0.69	0.7	0.31	1.3
		100	12.0	0.67	0.6	0.33	1.3
		120	11.9	0.66	0.6	0.34	1.1
		140	11.9	0.65	0.7	0.35	1.2
		160	11.7	0.65	0.6	0.35	1.3
		180	11.9	0.65	0.7	0.35	1.3
**Pc-ref** (95 μM)	645	40	12.3	1.00			1.2
		60	12.2	1.00			1.1
		80	12.2	1.00			1.1
		100	12.1	1.00			1.2
		120	12.1	1.00			1.3
		140	12.0	1.00			1.1
		160	12.0	1.00			1.1
		180	11.9	1.00			1.2

[a]Fluorescence lifetime (τ_i) and population (%) of each component, determined by the hydrostatic pressure single photon counting method in degassed solution at room temperature; λ_{ex} = 405 nm. [b]Monitoring wavelength.

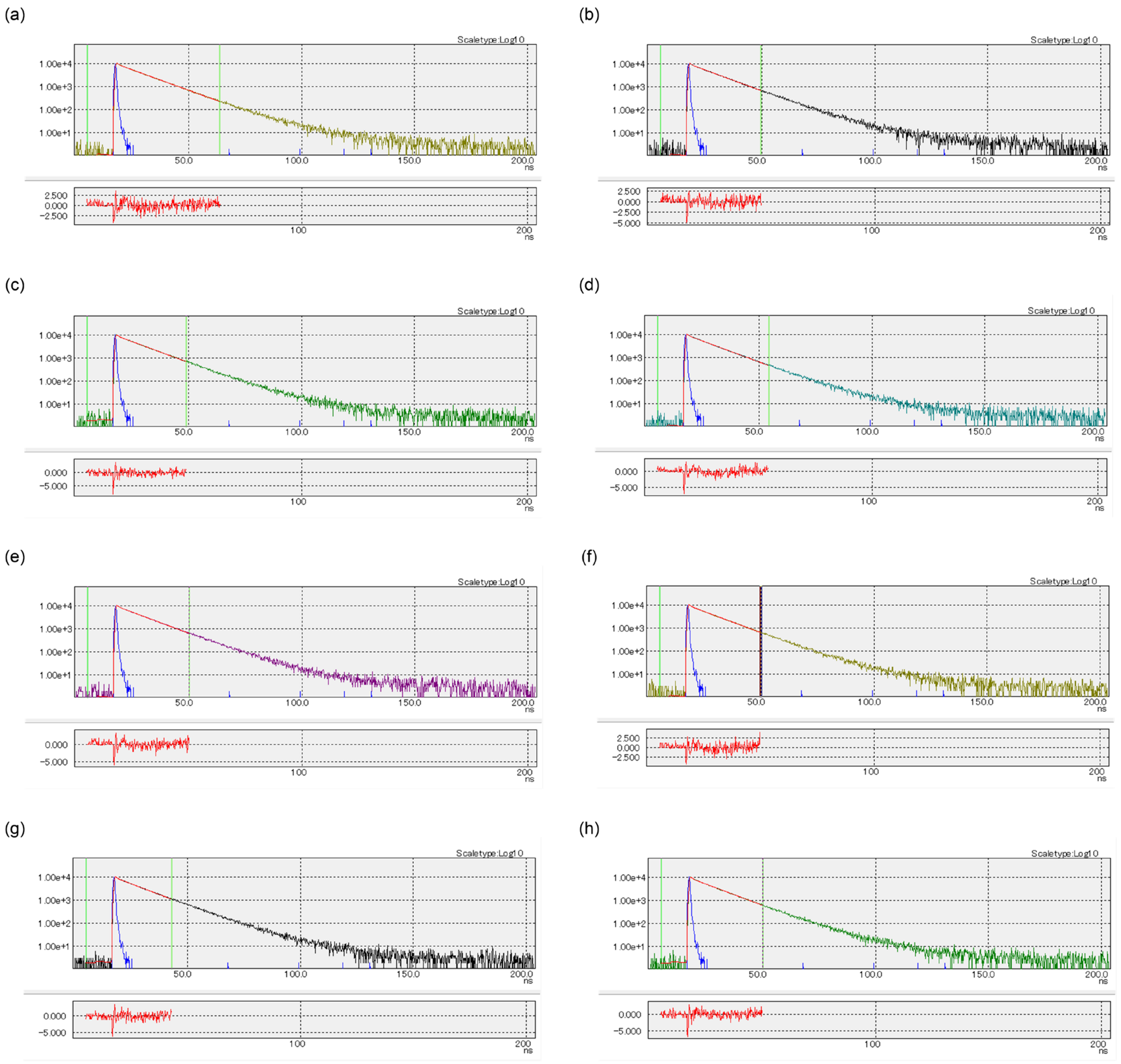

Figure S15. Time-correlated fluorescence decays of **Pc-BP-Pc** (77 μM) monitored at 655 nm at (a) 40, (b) 60, (c) 80, (d) 100, (e) 120, (f) 140, (g) 160, and (h) 180 MPa in THF at room temperature, measured in a high-pressure cell, where the colored, red, and blue lines represent the fluorescence decay, fitting result, and the instrument response function, respectively.

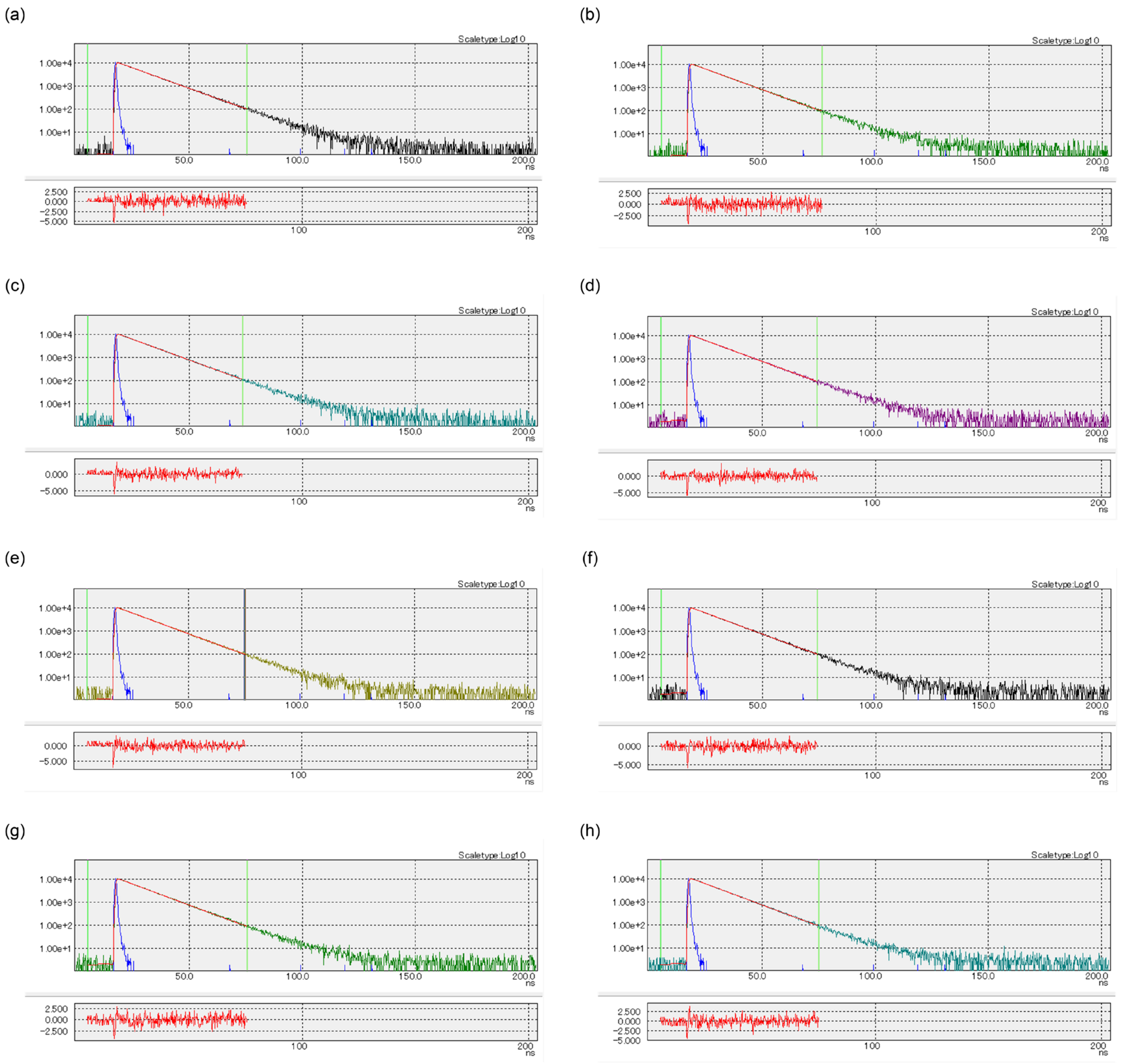

Figure S16. Time-correlated fluorescence decays of **Pc-ref** (95 μM) monitored at 645 nm at (a) 40, (b) 60, (c) 80, (d) 100, (e) 120, (f) 140, (g) 160, and (h) 180 MPa in THF at room temperature, measured in a high-pressure cell, where the colored, red, and blue lines represent the fluorescence decay, fitting result, and the instrument response function, respectively.

Table S5. Pressure Dependence of $k_{SF,app}$ of Pc-BP-Pc[a]

Pressure/MPa	$k_{SF,app}/10^9$ s^{-1} in toluene	$k_{SF,app}/10^9$ s^{-1} in MCH	$k_{SF,app}/10^9$ s^{-1} in THF
0.1	1.65	1.25	*b*
20	1.41	1.11	*b*
40	1.68	1.17	1.23
60	1.77	1.30	1.33
80	1.77	1.38	1.41
100	1.65	1.18	1.58
120	1.92	1.25	1.56
140	1.81	1.36	1.45
160	1.68	1.20	1.67
180	1.96	1.14	1.45

[a]Measured at 298 K. [b]Not determined.

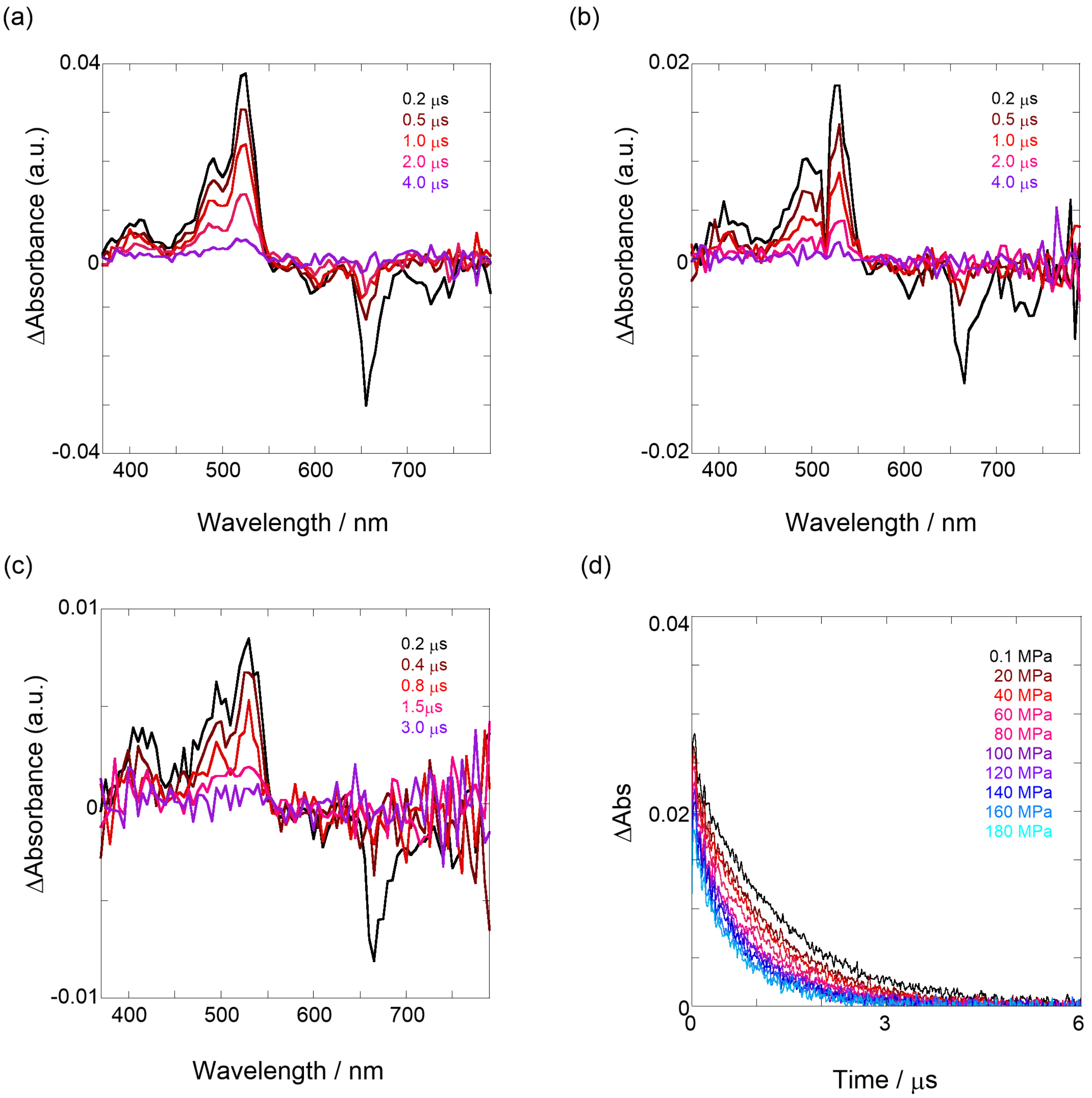

Figure S17. nsTA spectra (λ_{ex} = 532 nm) of **Pc-BP-Pc** (81 μM) in toluene at (a) 0.1, (b)160, and (c) 320 MPa and (d) nsTA decay profiles (λ_{ex} = 532 nm) of **Pc-BP-Pc** in MCH (84 μM, λ_{obs} = 516 nm) at 0.1, 20, 40, 60, 80, 100, 120, 140, 160, and 180 MPa (from black to sky blue) at room temperature, measured in a high-pressure cell.

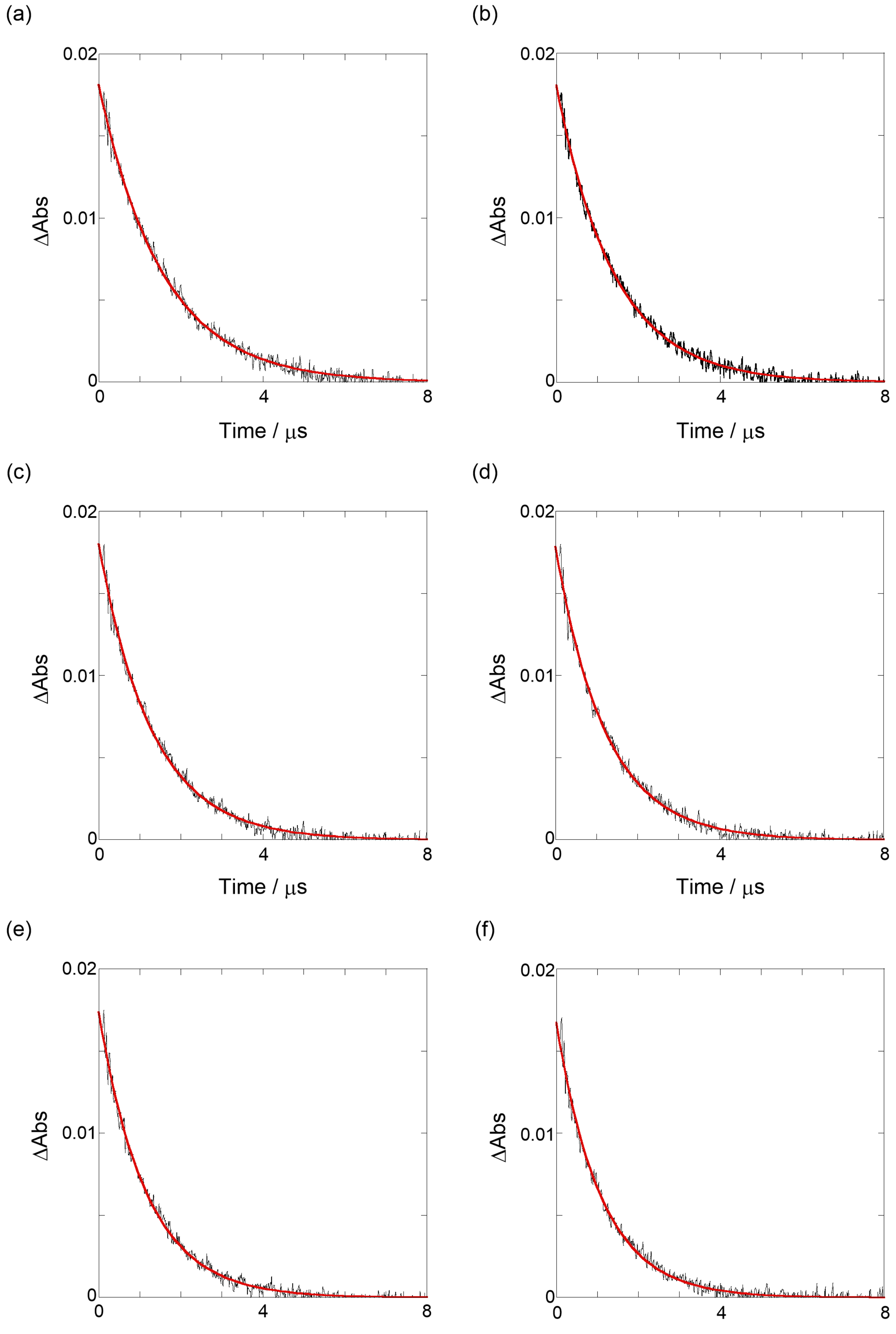

(a)
(b)
(c)
(d)
(e)
(f)
ΔAbs
0.02
0.01
0
0
4
8
Time / μs

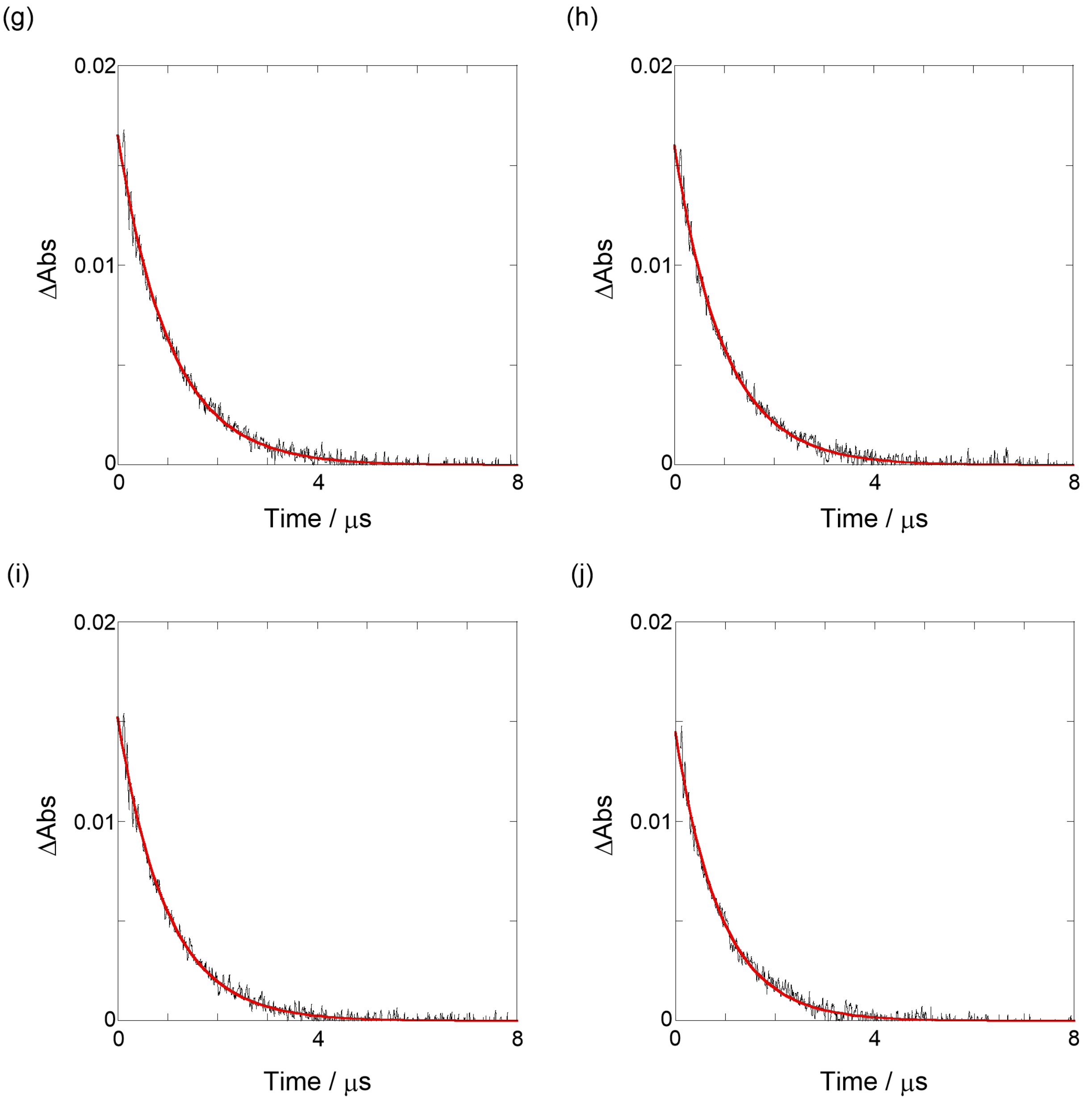

Figure S18. nsTA decay profiles (λ_{ex} = 532 nm, λ_{obs} = 525 nm) of **Pc-BP-Pc** (81 μM) in toluene at room temperature at (a) 0.1, (b) 20, (c) 40, (d) 60, (e) 80, (f) 100, (g) 120, (h) 140, (i) 160, and (j) 180 MPa, measured in a high-pressure cell, where the black and red lines represent the decay and fitting result, respectively.

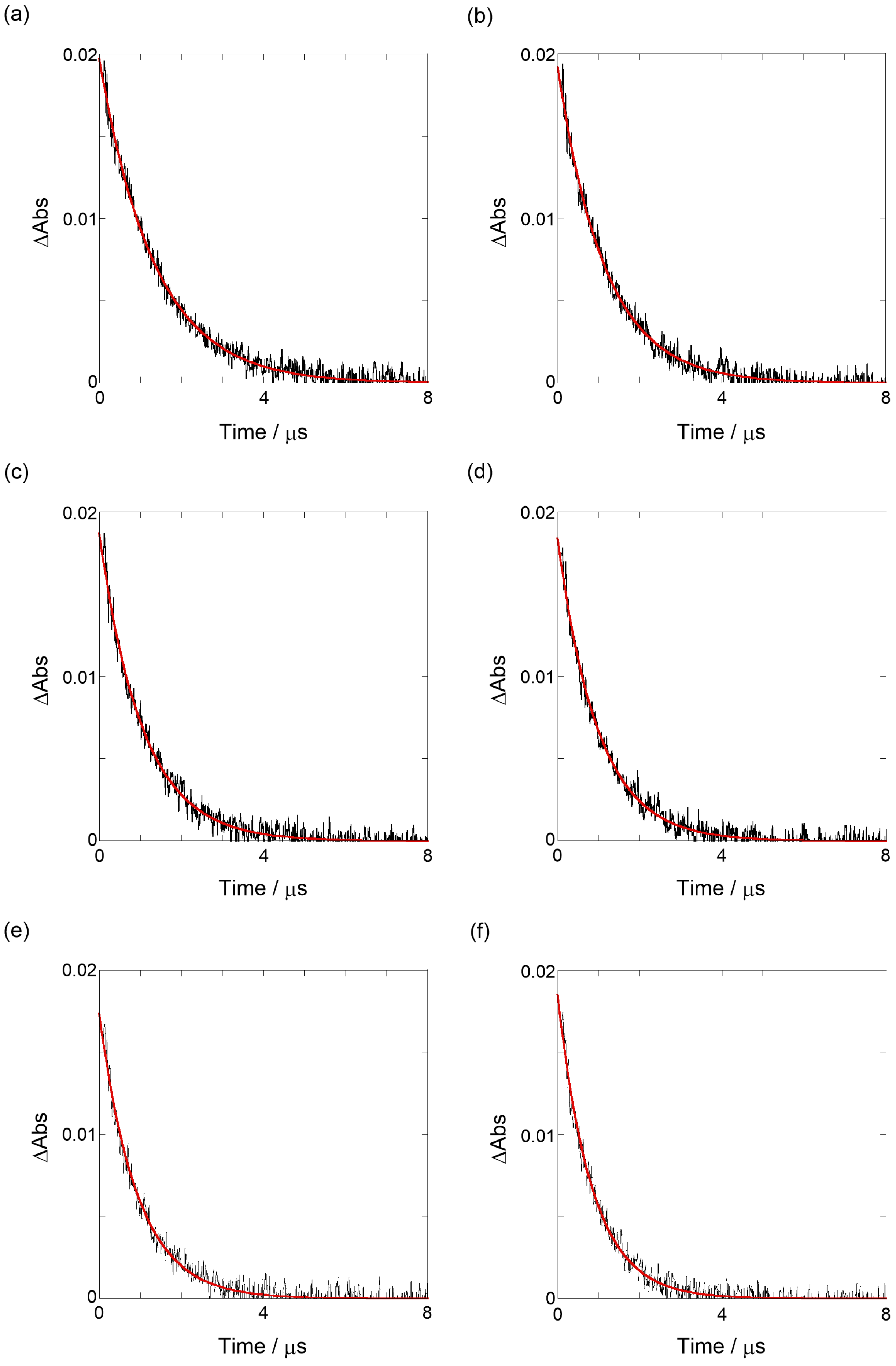
(a)
(b)
(c)
(d)
(e)
(f)
ΔAbs
0.02
0.01
0
Time / μs
0
4
8

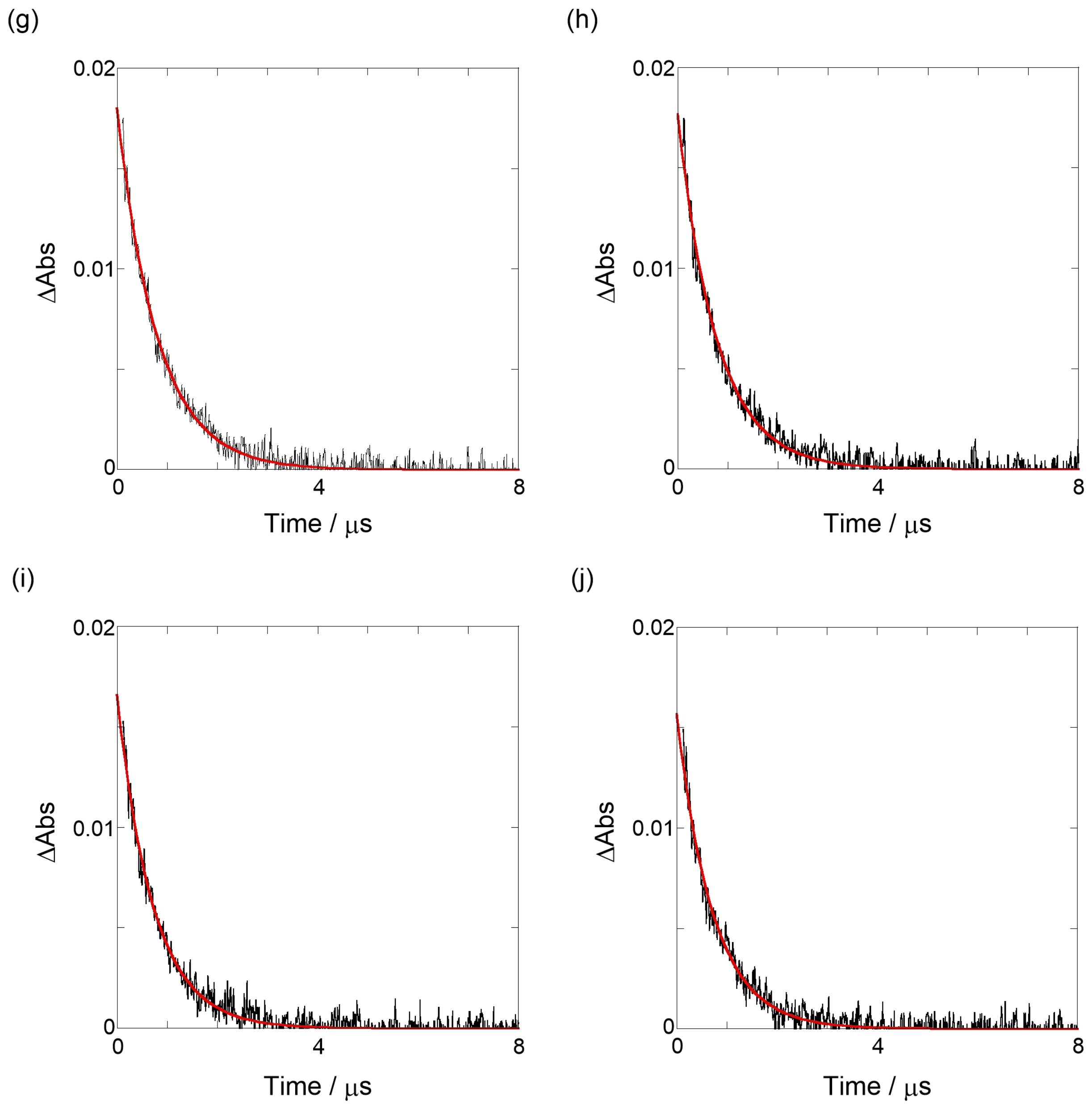

Figure S19. nsTA decay profiles (λ_{ex} = 532 nm, λ_{obs} = 520 nm) of **Pc-BP-Pc** (74 μM) in MCH at room temperature at (a) 0.1, (b) 20, (c) 40, (d) 60, (e) 80, (f) 100, (g) 120, (h) 140, (i) 160, and (j) 180 MPa, measured in a high-pressure cell, where the black and red lines represent the decay and fitting result, respectively.

Table S6. Triplet Lifetimes (τ_T) of Pc-BP-Pc in Toluene and MCH under Hydrostatic Pressure[a]

Pressure/MPa	τ_T/μs in toluene	τ_T/μs in MCH
0.1	1.56	1.35
20	1.40	1.15
40	1.30	1.05
60	1.22	0.98
80	1.16	0.92
100	1.09	0.83
120	1.04	0.80
140	0.99	0.78
160	0.98	0.72
180	0.91	0.72

[a]Measured at 298 K.

Table S7. Relative S_1 Quantum Yields (Φ_S) of Pc-BP-Pc (67 μM) in Toluene under Hydrostatic Pressure[a]

Pressure/MPa	d/g cm^{-3}	n_P	A_P[d]	D_P	Φ_S
0.1	0.862[b]	1.494	0.102	7770.8	0.0140
20	0.877[c]	1.503	0.103	7392.7	0.0134
40	0.890[c]	1.511	0.102	7098.5	0.0131
60	0.901[c]	1.518	0.102	6970.0	0.0130
80	0.911[c]	1.524	0.102	6858.7	0.0129
100	0.921[c]	1.530	0.102	6764.7	0.0128
120	0.930[c]	1.536	0.103	6726.6	0.0128
140	0.938[c]	1.540	0.102	6720.6	0.0129
160	0.945[c]	1.545	0.103	6747.8	0.0130
180	0.952[c]	1.549	0.103	6815.2	0.0131

[a]Measured at 298 K. [b]Ref. 47. [c]Ref. 48. [d]Extracted from Figure 2a.

Table S8. Relative S_1 Quantum Yields (Φ_S) of Pc-BP-Pc (87 μM) in MCH under Hydrostatic Pressure[a]

Pressure/MPa	d/g cm^{-3}	n_P	A_P[e]	D_P	Φ_S
0.1	0.765[b]	1.421	0.058	9643.0	0.0514
20	0.781[c]	1.430	0.057	9215.4	0.0503
40	0.794[c]	1.438	0.057	9070.1	0.0501
60	0.805[c]	1.444	0.057	8900.9	0.0496
80	0.816[c]	1.450	0.058	8794.5	0.0492
100	0.825[c]	1.456	0.058	8738.1	0.0488
120	0.834[c]	1.461	0.058	8875.1	0.0501
140	0.841[c]	1.466	0.058	8888.8	0.0499
160	0.846[d]	1.468	0.058	8913.7	0.0505
180	0.850[d]	1.471	0.058	8934.0	0.0507

[a]Measured at 298 K. [b]Ref. 47. [c]Ref. 49. [d]Extrapolated values. [e]Extracted from Figure S5a.

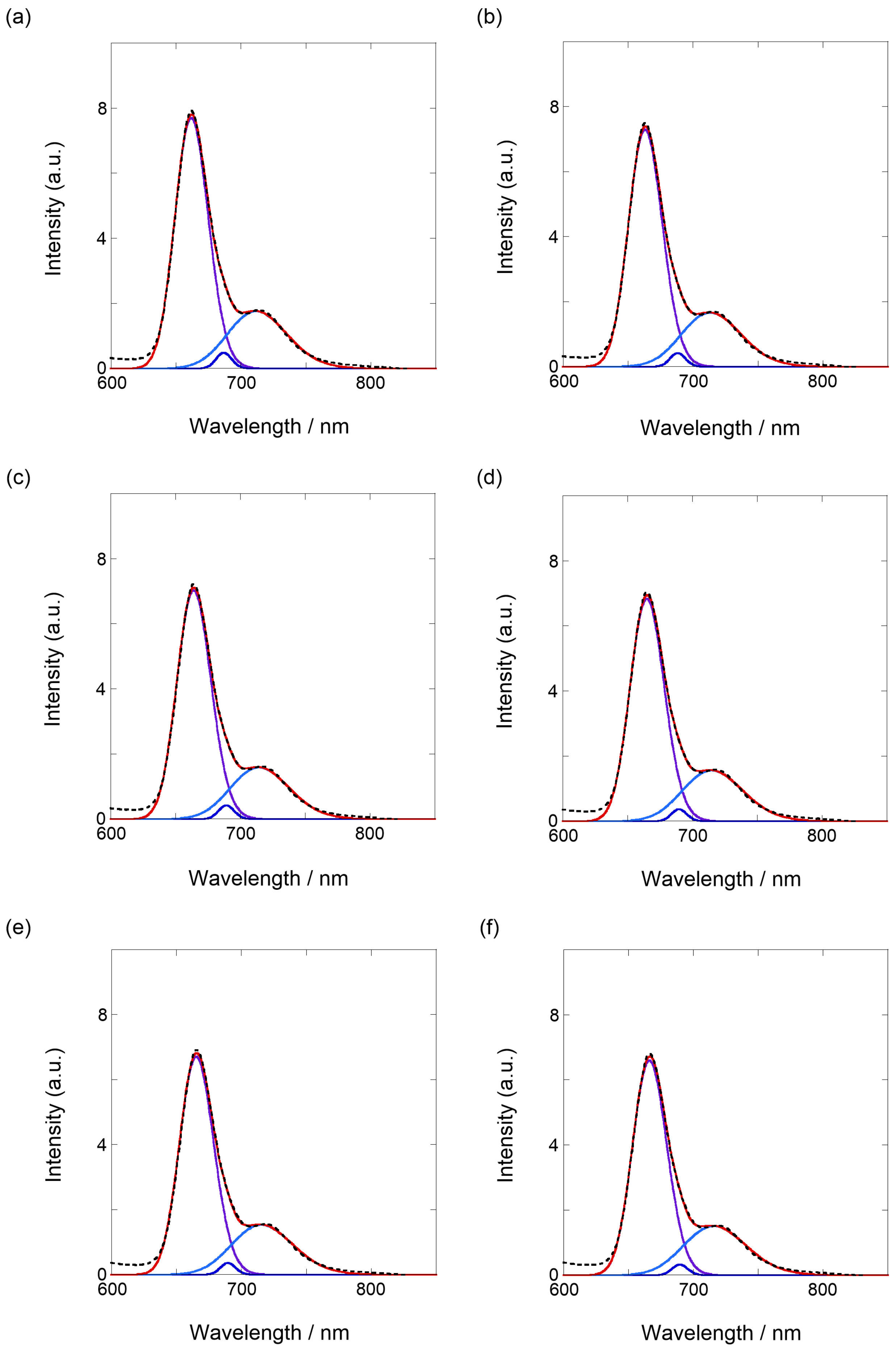
(a)
(b)
(c)
(d)
(e)
(f)
Intensity (a.u.)
Wavelength / nm
0
4
8
600
700
800

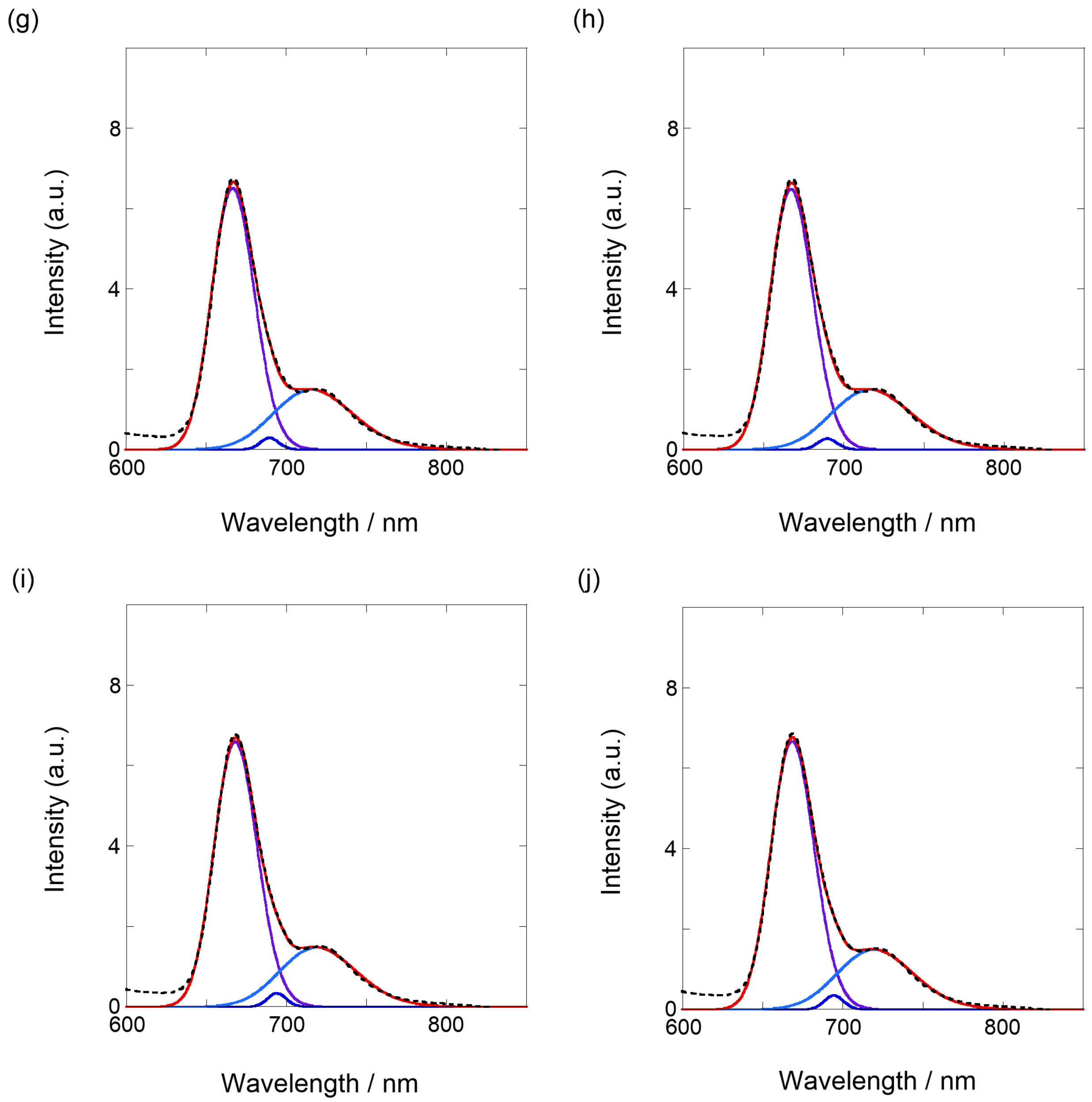

Figure S20. Fluorescence spectra (λ_{ex} = 580 nm, dotted lines), results of waveform separation spectra (purple, blue, and sky blue lines) and integrated spectra (red lines) of **Pc-BP-Pc** (67 μM) in toluene at (a) 0.1, (b) 20, (c) 40, (d) 60, (e) 80, (f) 100, (g) 120, (h) 140, (i) 160, and (j) 180 MPa.

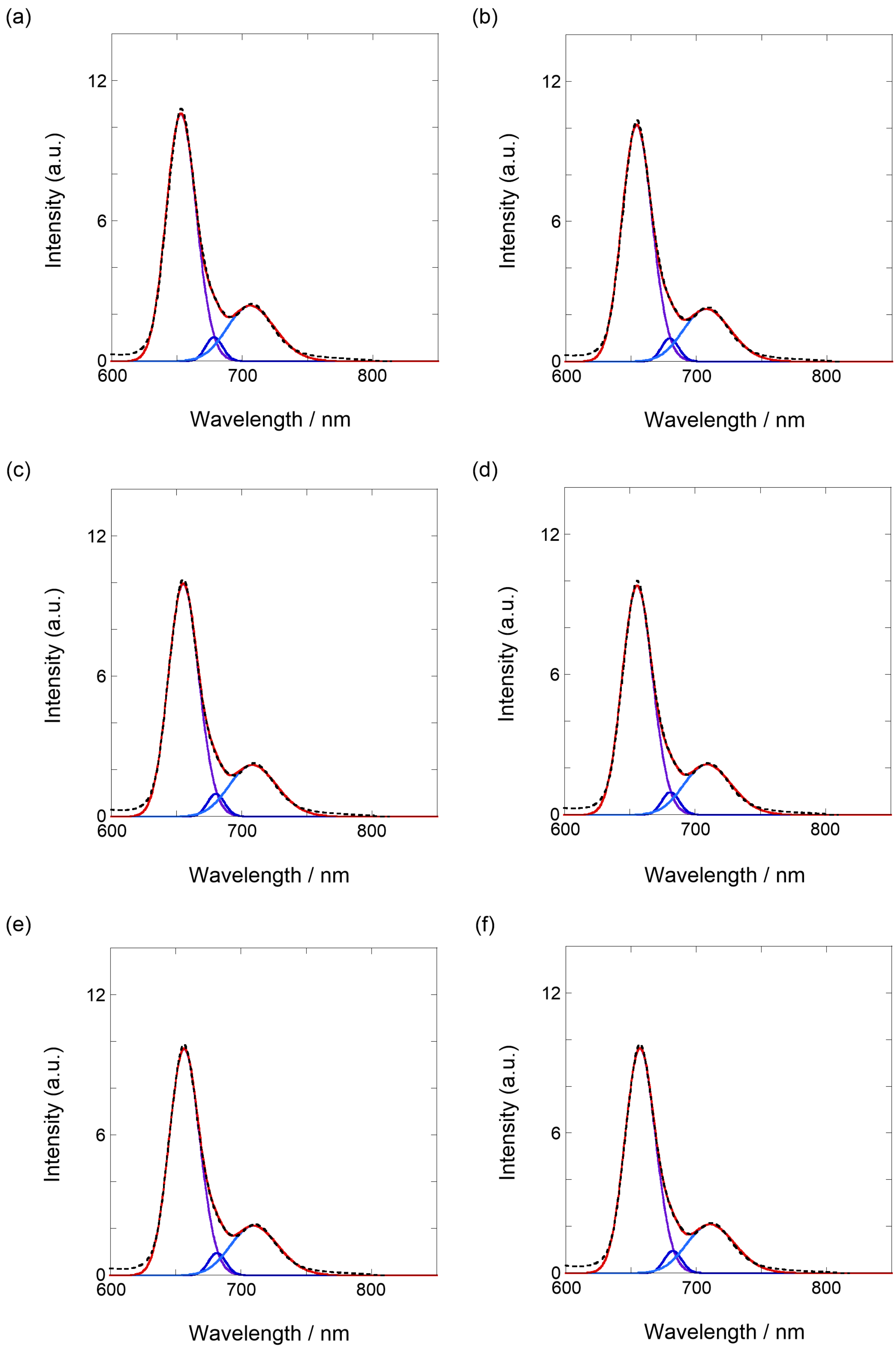
(a)
(b)
(c)
(d)
(e)
(f)
Intensity (a.u.)
Wavelength / nm
0
6
12
600
700
800

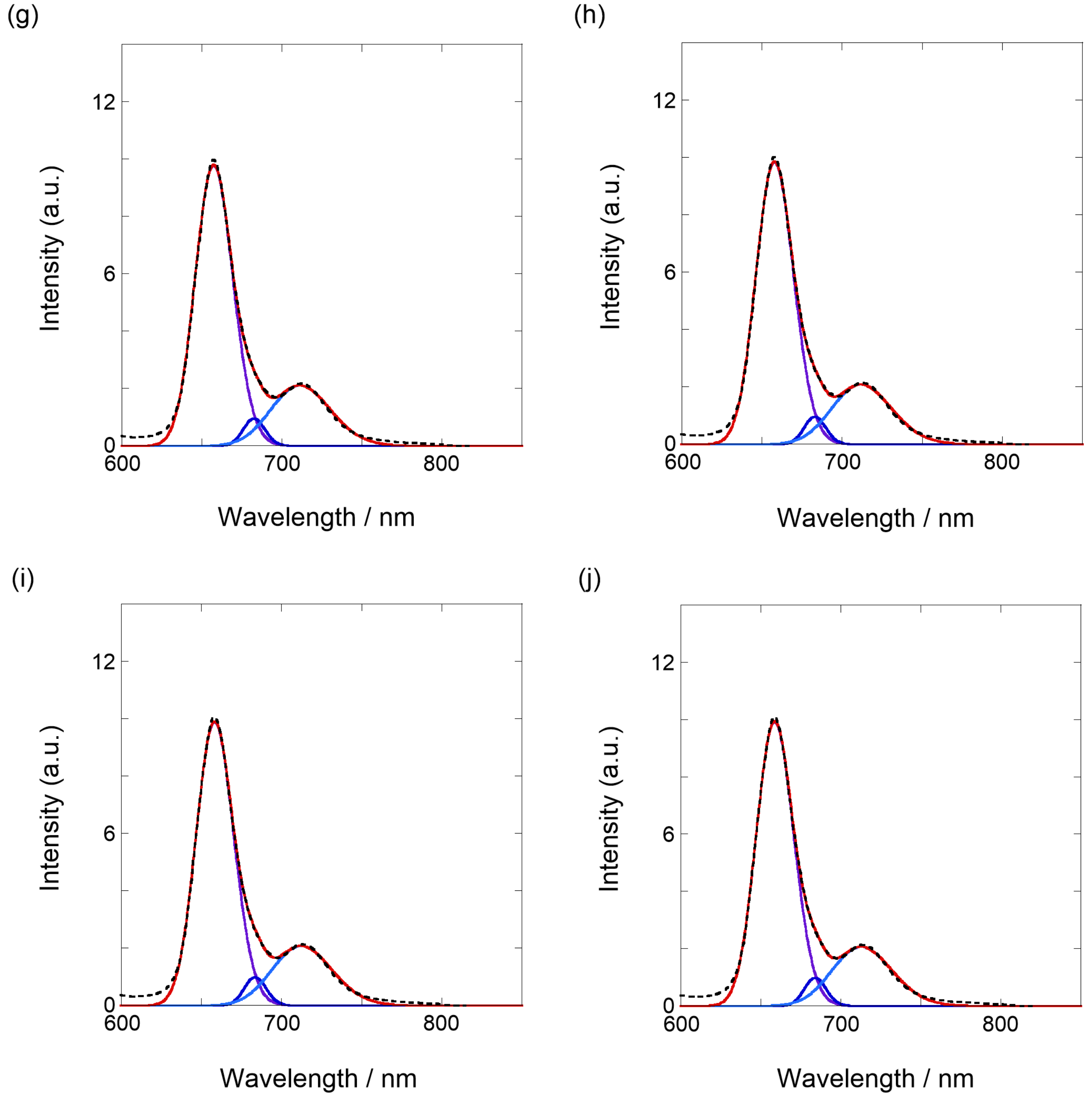

Figure S21. Fluorescence spectra (λ_{ex} = 576 nm, dotted lines), results of waveform separation spectra (purple, blue, and sky blue lines) and integrated spectra (red lines) of **Pc-BP-Pc** (87 μM) in MCH at (a) 0.1, (b) 20, (c) 40, (d) 60, (e) 80, (f) 100, (g) 120, (h) 140, (i) 160, and (j) 180 MPa.

Table S9. Fitting Parameters for Fluorescence Spectra of Pc-BP-Pc in Toluene

Pressure/MPa	a_1	b_1	c_1	a_2	b_2	c_2	a_3	b_3	c_3
0.1	7.696	15110	414.2	0.4788	14560	179.8	1.754	14050	633.1
20	7.301	15080	417.5	0.4175	14530	175.6	1.662	14020	631.4
40	7.026	15060	419.1	0.4161	14510	178.1	1.584	14000	622.6
60	6.840	15040	420.4	0.3640	14500	173.3	1.533	13990	639.9
80	6.711	15030	419.5	0.3572	14500	172.4	1.529	13980	649.3
100	6.594	15010	419.6	0.3247	14500	175.6	1.503	13970	660.5
120	6.512	15000	420.7	0.2888	14500	170.9	1.486	13970	677.1
140	6.488	14990	420.3	0.2734	14500	178.5	1.485	13960	684.2
160	6.591	14970	425.0	0.3331	14410	170.1	1.471	13910	645.3
180	6.672	14960	425.0	0.3437	14400	172.6	1.481	13900	641.6

Table S10. Fitting Parameters for Fluorescence Spectra of Pc-BP-Pc in MCH

Pressure/MPa	a_1	b_1	c_1	a_2	b_2	c_2	a_3	b_3	c_3
0.1	10.580	15310	380.2	1.0260	14740	198.6	2.371	14120	509.0
20	10.130	15280	380.9	0.9928	14710	202.0	2.252	14130	506.3
40	9.963	15260	381.6	0.9673	14700	200.6	2.203	14120	509.0
60	9.798	15250	381.4	0.9651	14680	202.4	2.151	14100	506.5
80	9.694	15230	381.4	0.9496	14670	201.9	2.113	14090	507.7
100	9.632	15220	381.3	0.9492	14660	202.8	2.080	14070	511.9
120	9.786	15210	381.5	0.9499	14650	203.3	2.101	14060	514.4
140	9.837	15200	381.3	0.9546	14640	203.1	2.079	14050	514.8
160	9.890	15190	380.1	0.9829	14630	206.2	2.075	14040	514.3
180	9.920	15180	380.2	0.9824	14620	205.4	2.064	14030	517.0

Table S11. Pressure Dependence of Correlated Triplet Pair Quantum Yields (Φ_{TT}) of Pc-BP-Pc[a]

Pressure/MPa	Φ_{TT} in toluene	Φ_{TT} in MCH
0.1	0.955	0.950
20	0.947	0.943
40	0.955	0.946
60	0.958	0.951
80	0.957	0.954
100	0.955	0.947
120	0.961	0.951
140	0.959	0.959
160	0.956	0.955
180	0.962	0.947

[a]Measured at 298 K.

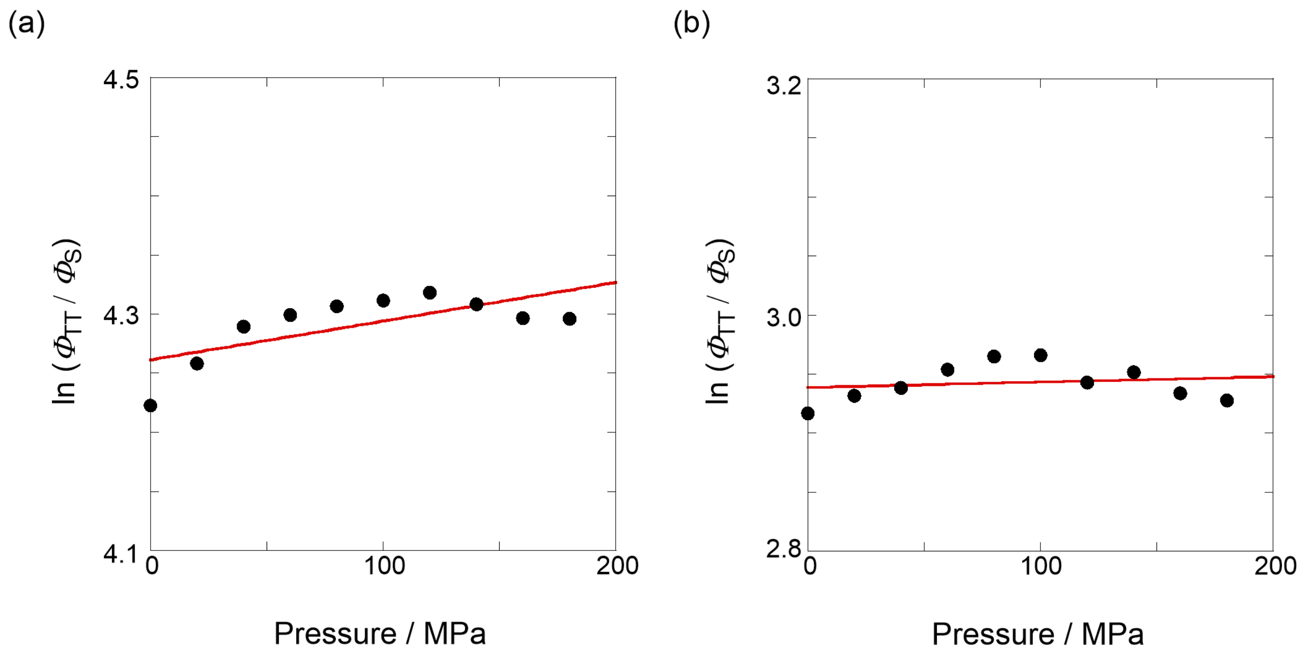

Figure S22. Pressure dependence of relative abundance ratio (Φ_{TT}/Φ_S) of **Pc-BP-Pc** in (a) toluene (r = 0.685) and (b) MCH (r = 0.169) at room temperature.

Table S12. Triplet Quantum Yields (Φ_T) of Pc-BP-Pc in Toluene under Hydrostatic Pressure

Pressure/MPa	$\Delta A_{\mathbf{Pc-BP-Pc}}{}^{a}$	$\Delta A_{\mathrm{ZnTPP}}{}^{b}$	$A_{\mathbf{Pc-BP-Pc}}$	A_{ZnTPP}	$\Phi_{\mathrm{T},\mathbf{Pc-BP-Pc}}$
0.1	0.026	0.078	0.054[a]	0.254[b]	1.76
20	0.025	0.077	0.055[c]	0.260[d]	1.70
40	0.025	0.075	0.056[c]	0.259[d]	1.69
60	0.024	0.072	0.056[c]	0.258[d]	1.67
80	0.022	0.074	0.057[c]	0.258[d]	1.50
100	0.021	0.071	0.057[c]	0.258[d]	1.48
120	0.020	0.070	0.059[c]	0.257[d]	1.40
140	0.019	0.069	0.059[c]	0.259[d]	1.37
160	0.018	0.070	0.059[c]	0.258[d]	1.24
180	0.017	0.069	0.059[c]	0.252[d]	1.15

[a]**Pc-BP-Pc** (81 μM) in toluene. [b]**ZnTPP** (247 μM) in toluene. [c]Corrected by Figure 2a. [d]Corrected by Figure S23b.

Table S13. Triplet Quantum Yields (Φ_T) of Pc-BP-Pc in MCH under Hydrostatic Pressure

Pressure/MPa	$\Delta A_{\mathbf{Pc-BP-Pc}}{}^{a}$	$\Delta A_{\mathrm{ZnTPP}}{}^{b}$	$A_{\mathbf{Pc-BP-Pc}}$	A_{ZnTPP}	$\Phi_{\mathrm{T},\mathbf{Pc-BP-Pc}}$
0.1	0.028	0.078	0.090[a]	0.254[b]	1.12
20	0.026	0.077	0.090[c]	0.260[d]	1.10
40	0.026	0.075	0.091[c]	0.259[d]	1.08
60	0.025	0.072	0.093[c]	0.258[d]	1.05
80	0.023	0.074	0.094[c]	0.258[d]	0.95
100	0.022	0.071	0.098[c]	0.258[d]	0.92
120	0.022	0.070	0.098[c]	0.257[d]	0.91
140	0.021	0.069	0.100[c]	0.259[d]	0.88
160	0.020	0.070	0.100[c]	0.258[d]	0.81
180	0.018	0.069	0.101[c]	0.252[d]	0.72

[a]**Pc-BP-Pc** (84 μM) in MCH. [b]**ZnTPP** (247 μM) in toluene. [c]Corrected by Figure S5a. [d]Corrected by Figure S23b.

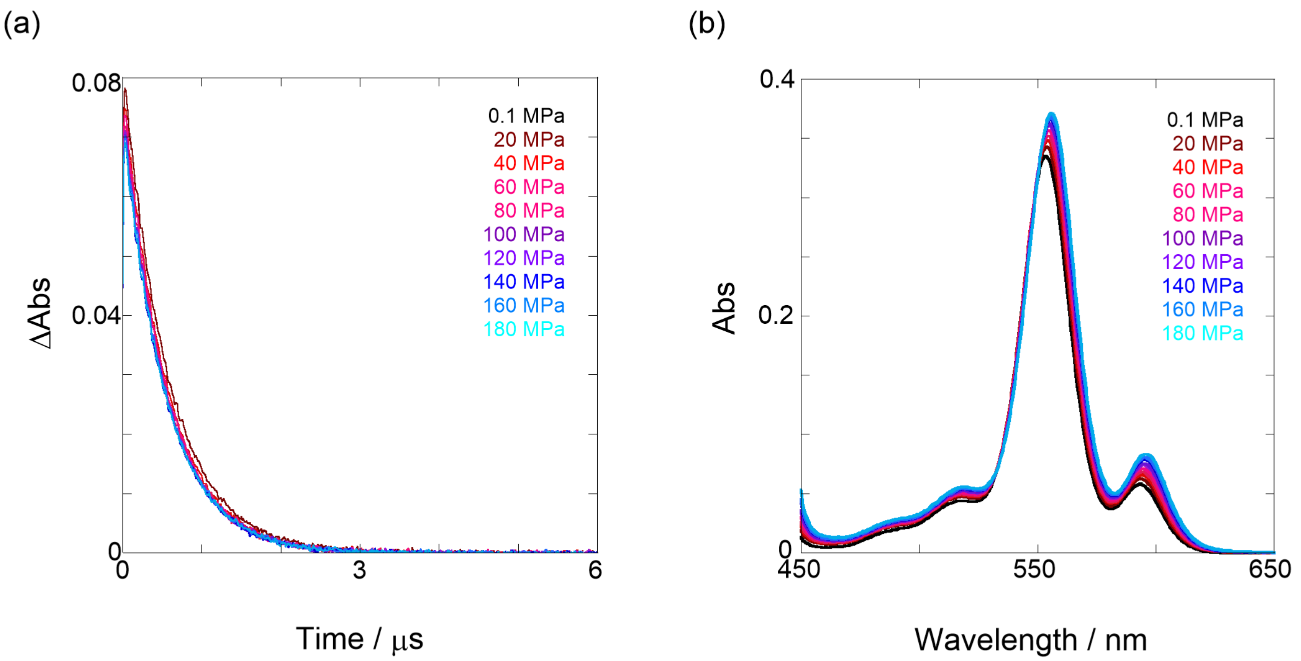

Figure S23. (a) nsTA decay profiles (λ_{ex} = 532 nm, λ_{obs} = 470 nm) and (b) UV/vis spectra of **ZnTPP** (113 μM) in toluene at room temperature at 0.1, 20, 40, 60, 80, 100, 120, 140, 160, and 180 MPa (from black to sky blue), measured in a high-pressure cell.

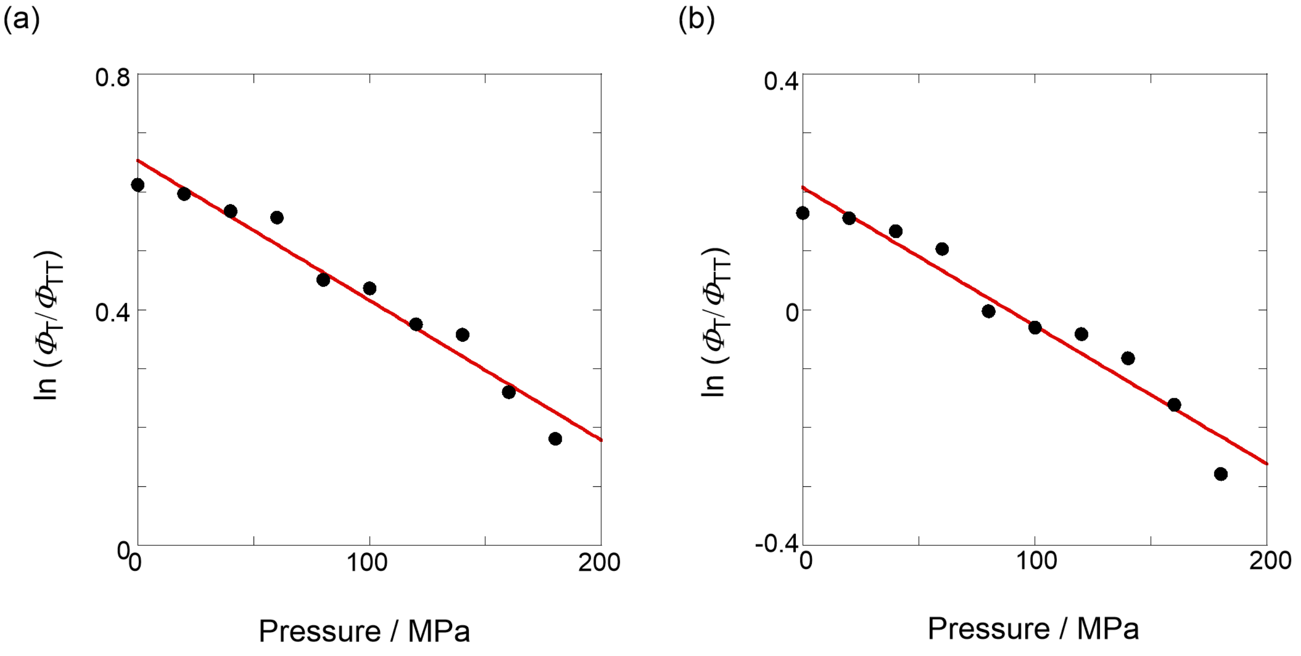

Figure S24. Pressure dependence of relative abundance ratio (Φ_T/Φ_{TT}) of **Pc-BP-Pc** in (a) toluene (r = 0.979) and (b) MCH (r = 0.972) at room temperature.

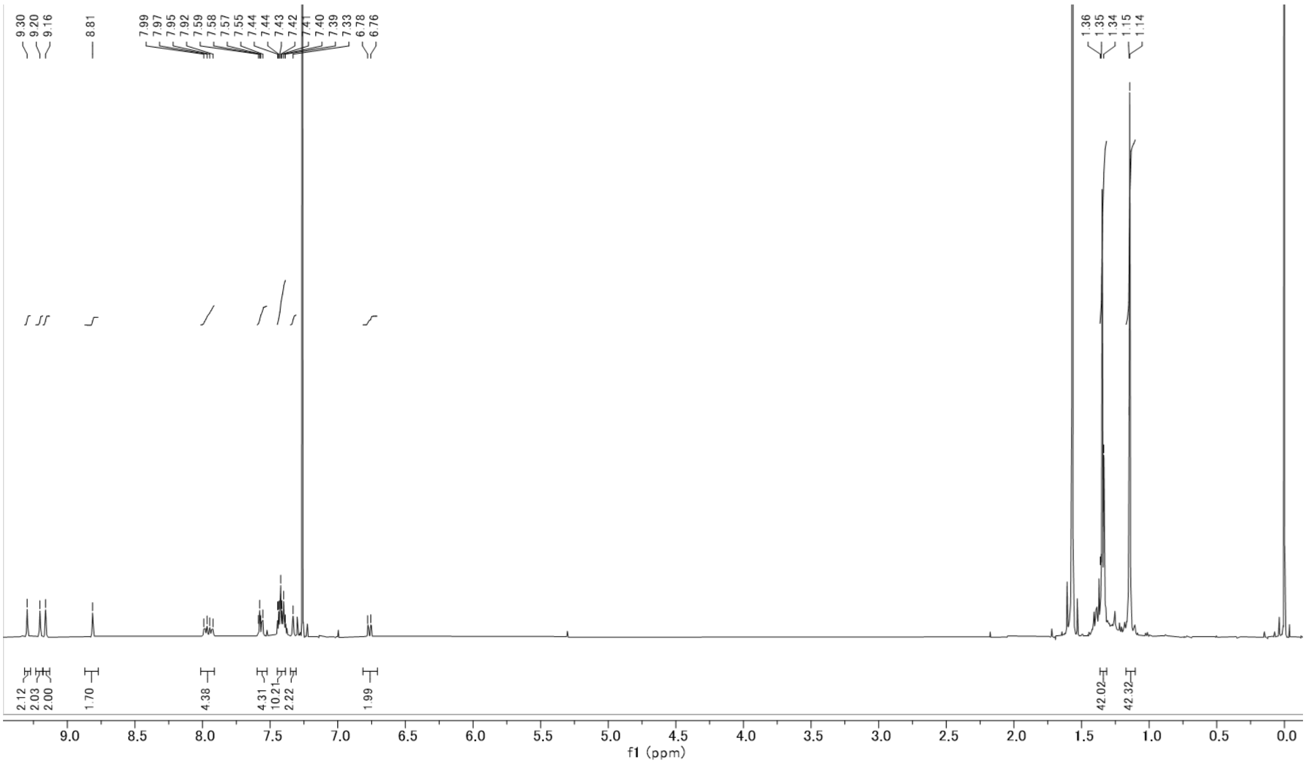

Figure S25. ^{1}H NMR spectrum of **Pc-BP-Pc** in $CDCl_3$.

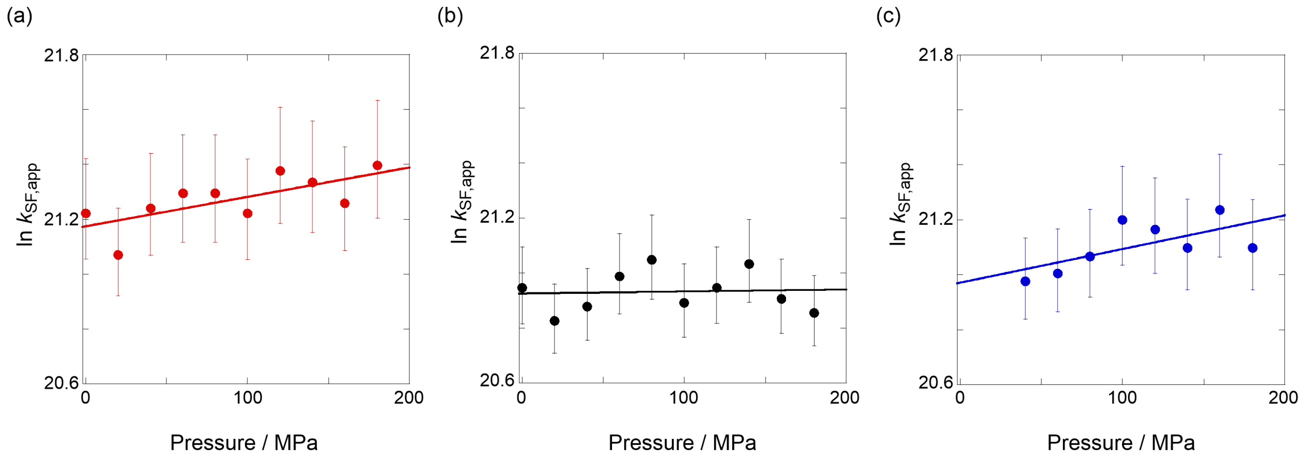

Figure S26. Pressure dependence of SF rate constants ($k_{SF,app}$) of **Pc-BP-Pc** in toluene (red, $r = 0.655$), THF (blue, $r = 0.706$), and MCH (black, $r = 0.059$) at room temperature.

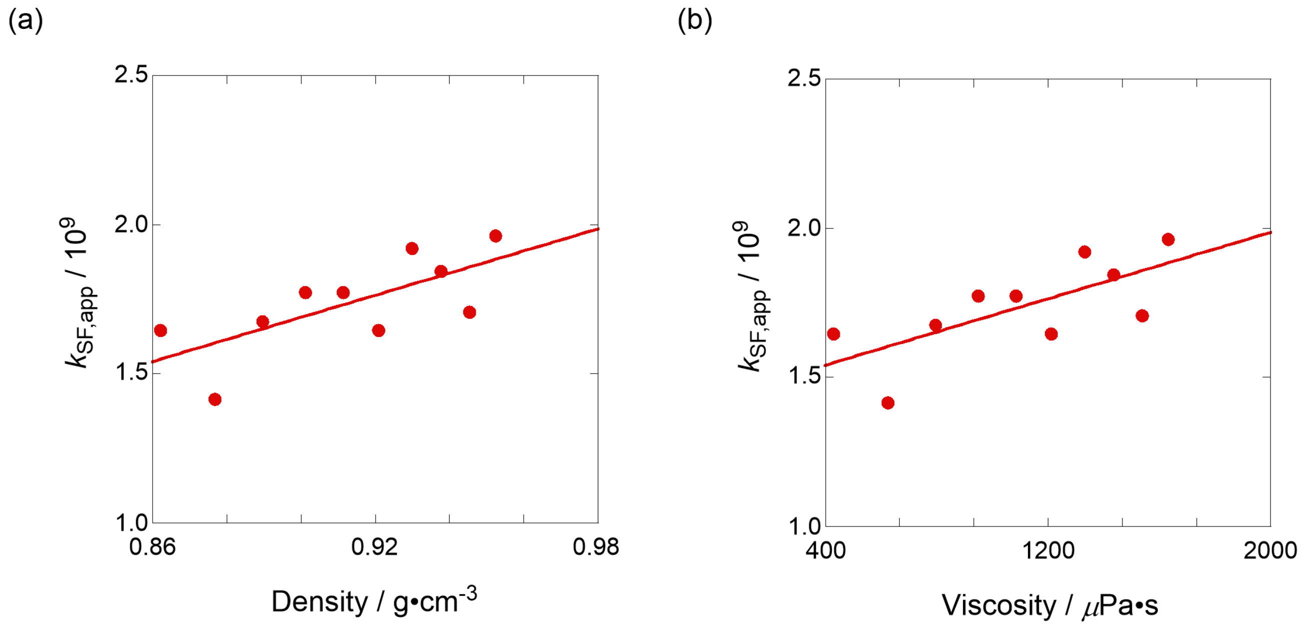

Figure S27. Pressure dependence of SF rate constants ($k_{SF,app}$) of **Pc-BP-Pc** as a function of (a) toluene density and (b) toluene viscosity at room temperature.
